# Smart home soundscape: constructing a perceptual model with qualitative and quantitative methods

**DOI:** 10.3389/fpsyg.2026.1803294

**Published:** 2026-06-05

**Authors:** Zhongzhe Li, Menghan Li, Meihui Ba, Wenlan Xu, Jian Kang

**Affiliations:** 1 Pan Tianshou College of Architecture, Art and Design, Ningbo University, Ningbo, China; 2 Institute for Environmental Design and Engineering, The Bartlett, University College London (UCL), London, United Kingdom

**Keywords:** grounded theory, mixed-methods, perceptual model, smart home soundscape, virtual reality

## Abstract

The proliferation of smart technology has led to the diversification and randomization of indoor sound sources. This necessitates a more nuanced consideration of user subjective experiences in indoor soundscape design. Consequently, identifying the perceptual dimensions of smart home soundscapes has become a critical issue in indoor sound environment design. Accordingly, this study aimed to identify the perceptual dimensions of smart home soundscapes, using a mixed-methods approach that combined qualitative and quantitative techniques. First, perceptual indicators were extracted based on grounded theory. Subsequently, virtual reality (VR) technology was used to simulate 12 different sound sources within three typical scenarios (Rest/ Entertainment/ Work). Quantitative perceptual data were collected from 120 participants in laboratory settings. Finally, factor analysis was applied to extract the perceptual dimensions of both the sound sources and sound environment. The results indicated the following: (1) Interview data culminated in 29 evaluation indicators for sound sources and 21 indicators for the sound environment. (2) The perceptual dimensions of the sound sources included comfort, informativeness, pleasantness, clarity, and redundancy. (3) The overall perception of the sound environment can be summarized as comfort and recognisability. Furthermore, the study discussed perceptual differences across various sound sources and scenario types through an analysis of variance (ANOVA) and principal component distributions. The conclusions provide a theoretical framework for indoor soundscape design and offer design implications for the acoustic optimisation of smart home products.

## 1 Introduction

The rapid growth in the global adoption of smart home products has been propelled by advancements in internet of things (IoT), artificial intelligence (AI), and low-power sensor chips (Chakraborty et al., 2023), with the industry's value expanding at a compound annual growth rate of 27% and projected to reach 13 trillion USD by 2030 (Rock et al., 2024; Grand View Research, 2025). The applications of smart sensors and control systems have driven functional diversification in smart appliances, spanning domains such as entertainment, environmental regulation, and automated monitoring (Marikyan et al., 2019). Consequently, the sound environment in smart homes exhibits distinct characteristics compared to traditional settings. First, the proliferation of interactive technologies and AI algorithms enables autonomous operation of devices (Li and Wu, 2022; Xu et al., 2024), complicating both sound quality control and sound environmental complexity (Alkan et al., 2023; Shabbir et al., 2025). Second, users' adoption of smart homes inherently reflects the acceptance of a smart lifestyle, wherein subjective perception, psychological needs, and behavioral responses become critical determinants of acoustic experience (Sadikoglu-Asan, 2022; Zhang et al., 2025). This acknowledgment of “agency” in smart systems fundamentally alters psychological expectations toward the sound environment relative to conventional homes. Thus, soundscape design for smart homes must embrace a human-centric principle that prioritizes users' subjective perceptions to ensure the optimal suitability of indoor sound environments for occupants.

The term soundscape was first introduced by Southworth in 1967 and was later popularized and further developed by Schafer. It was subsequently defined by the International Organization for Standardization (ISO) as “the sound environment as perceived, experienced, or understood by a person or people in context” (ISO/DIS, 2014). Unlike traditional acoustics, which focuses on physical measurements, soundscapes emphasize people's subjective perceptions and cognitive responses to sound in specific contexts (Kang and Schulte-Fortkamp, 2016). Early soundscape research focused on the sound environments of outdoor public open spaces. Studies on indoor soundscapes have also concentrated on the sound environment design of large public spaces such as underground stations (Kang et al., 2016; Yilmazer and Bora, 2017). In recent years, indoor soundscape research has mostly concentrated on the interior spaces of public buildings that are closely related to people's daily lives, including high-occupancy spaces such as offices (Ma and Shu, 2018; Rachman et al., 2024), healthcare facilities (Mackrill et al., 2013; Xie et al., 2025) and educational institutions (Pellegatti et al., 2023; Kurukose Cal et al., 2024). In contrast, research related to the domestic environment remains relatively limited, owing to its high privacy and complex personalized needs. Existing studies have mainly focused on the impact of traffic and neighborhood noise on indoor soundscapes (Berglund and Nilsson, 2002; Lu et al., 2023; Chen et al., 2024). Torresin et al. (2020) developed a principal component model for indoor soundscapes in residential spaces and identified two primary dimensions: comfortable and full of content. Chen et al. (2023) constructed an evaluation model for a healthy indoor acoustic environment in residential buildings, defining four characteristics of a healthy home sound environment: attachment, privacy, autonomy, and controllability. However, the increasing intelligence of domestic environments is likely to influence these perceptual models: (1) The proliferation of remote communication, online learning, and smart logistics has significantly diversified the types of activities conducted at home (Li et al., 2025; Lin et al., 2024); (2) The COVID-19 pandemic has further accelerated this trend, leading to extended time spent at home and heightened expectations for indoor sound environmental quality (Torresin et al., 2021, 2022); (3) More importantly, a paradigm shift in cognition is occurring—smart technologies are reshaping the relationship between people and space. The deep integration of smart home systems blurs the boundaries of traditional indoor spatial attributes, and the distinctions between home and outdoors (e.g., remote work blurring the physical boundaries between work and life), reality and virtuality (e.g., immersive experiences created by VR technologies), and humans and objects (e.g., human-like interactions exhibited by smart home devices) are gradually dissolving. These three changes suggest that, driven by intelligent transformations and evolving lifestyles, perceptual models of indoor soundscapes urgently need to be re-examined and systematically studied.

Conventional approaches to indoor sound environment control have predominantly relied on regulating physical acoustic parameters, such as sound pressure level and reverberation time, to mitigate noise or optimize sound energy distribution (ISO, 2009; Kuttruff, 2016). The establishment of correlations between physical indicators and auditory perception has further promoted the application of psychoacoustic parameters, such as loudness, speech intelligibility, and other related measures (Zwicker and Fastl, 2013; Shtrepi et al., 2024). Nonetheless, these methodologies often depend on static or equivalent condition measurements and simulations, thereby underestimating the immediacy and dynamism of human auditory perception in real-world settings (Hossain et al., 2025). Cognitive psychology posits that the auditory cognitive process unfolds in three approximate stages. First, listeners recognize the physical attributes of individual sound sources, such as spectral features and spatial location (Huang and Elhilali, 2017). Second, acoustic events are organized and semantically interpreted (Broderick et al., 2019; Kothinti and Elhilali, 2023). Finally, multiple sources are integrated into a coherent holistic perception (Bizley and Cohen, 2013). This framework highlights the bottom-up processing mechanisms of the auditory system. Concurrently, top-down cognitive mechanisms also operate, as exemplified by the cocktail party effect, wherein attention is selectively directed toward a single sound source in a complex sound environment (Bronkhorst, 2015). Substantial evidence indicates that auditory cognition is highly sensitive to sound source characteristics, temporal patterns, and contextual congruence (Shamma, 2001; Moore, 2012; Braat-Eggen et al., 2019). This suggests that the transition from individual sound sources to the overall sound environment is not a mere linear aggregation. Instead, it involves intricate non-linear interactions between source-specific and environment-wide perception, calling for integrated perceptual research at the psychological level. This complex relationship has been corroborated in automotive cabin research—a similarly intricate “indoor” space—where studies address not only physical noise control but also examine, through subjective assessment and psychoacoustic parameters, the interplay between discrete sound events and the holistic cabin soundscape experience (Swart and Bekker, 2019; Kim et al., 2022; Song et al., 2024). Insights from product sound quality to environmental acoustics collectively demonstrate that perceptions of sound sources and sound environments are not linearly correlated, but emerge from interactions across varying spatiotemporal scales and contextual scenarios. Thus, research on smart home soundscapes should emphasize both the distinctions and interrelationships between sound sources and environments.

Methodologies for investigating sound environment perception encompass qualitative, quantitative, and hybrid approaches, with the selection often determined by research aims, contextual factors, and ecological validity (Aletta et al., 2016). Qualitative inquiry, grounded in sociological and anthropological paradigms, prioritizes contextual narration and the construction of meaning, employing techniques such as focus groups (Torresin et al., 2024), narrative interviews (Liu and Kang, 2016), open-ended descriptions during soundwalks (Davies et al., 2013), and behavioral observations to gather rich empirical material. Researchers typically engage in inductive and semantic analyses of textual or verbal accounts to derive key concepts and categories, culminating in a semantic framework for interpreting soundscape experiences. Conversely, quantitative studies use semantic differential scales or empirical measurements to transform perceptions into statistically tractable numerical data. For instance, Axelsson et al. (2010) operationalised affective experience along the “Pleasantness–Eventfulness” dimensions, whereas other investigations have integrated physiological indicators (e.g., electrodermal activity, heart rate variability) to corroborate subjective perceptual variations with objective physiological responses (Li et al., 2021). Quantitative methods have been extensively applied in soundscape research because of their ability to provide quantifiable, comparable, and generalizable analytical frameworks (Pheasant et al., 2010; Wilford et al., 2021; Mitchell et al., 2022). Hybrid methods, which blend qualitative and quantitative techniques, harness the advantages of both paradigms and facilitate the deep interpretation of subjective experience alongside systematic data validation. Examples include acquiring qualitative soundscape descriptions via interviews or open-ended questionnaires and then applying factor analysis or structural equation modeling to quantify the structure and weighting of perceptual dimensions (Acun and Yilmazer, 2019; Jia et al., 2020; Cal et al., 2025). Considering that users' basic perceptions of “smart sounds” are not yet well-defined, and the necessity to balance measurability and generalizability of research results, smart home soundscape research is advised to employ a mixed-methods strategy, elucidating users' subjective perceptions through semantic analysis, while leveraging statistical techniques to refine and validate core dimensions, ultimately enabling the construction of an emotion-oriented soundscape model for smart environments.

**Figure 1:**
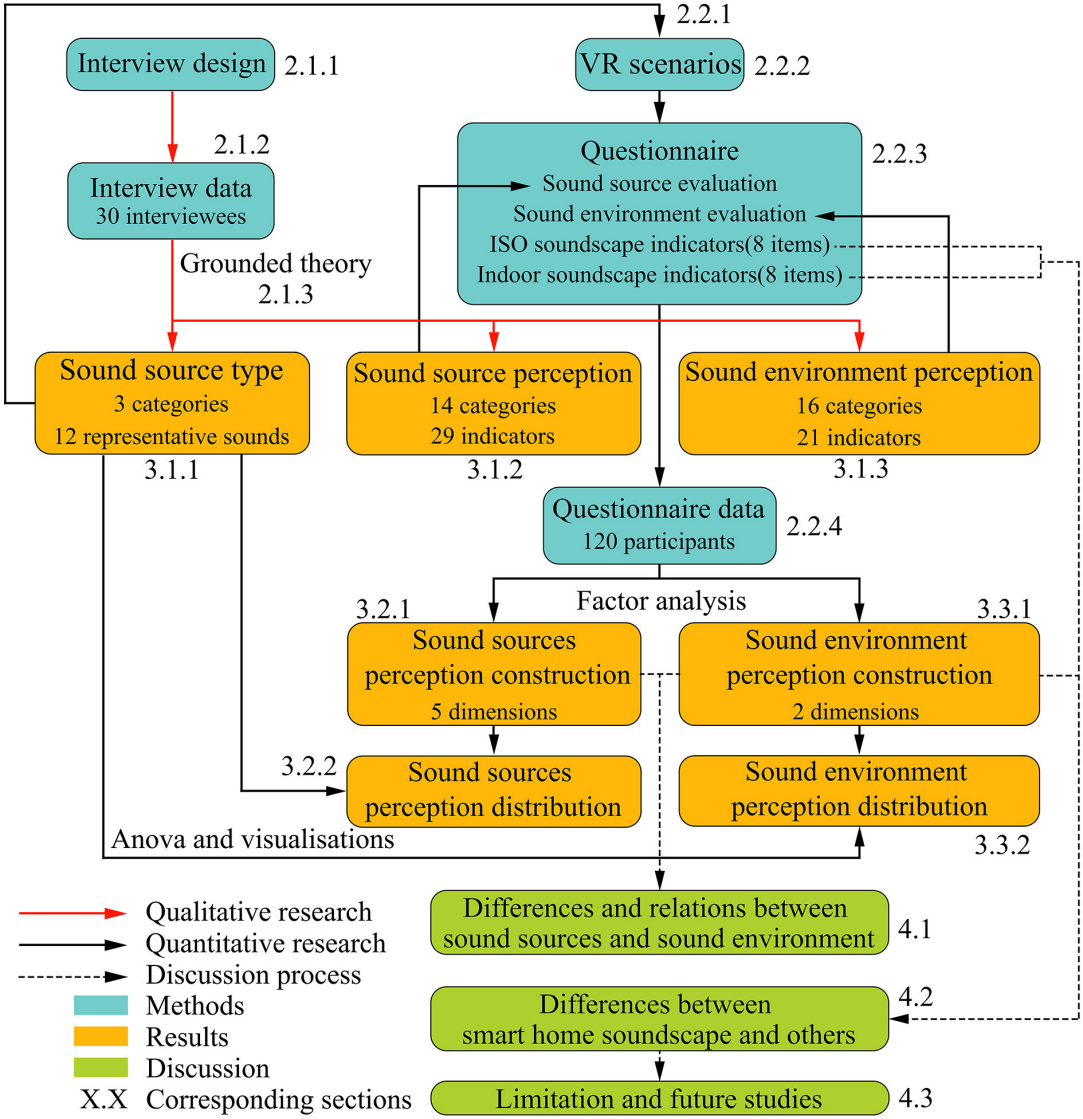
Research framework.

In summary, this study adopts a mixed-methods approach that combines qualitative and quantitative research. First, perceptual indicators were extracted through qualitative coding of the interview data. Subsequently, experiments and subjective questionnaires were conducted to construct a perceptual model of smart home soundscapes. This study aimed to identify the perceptual dimensions of smart home soundscapes, using a mixed-methods approach that combined qualitative and quantitative techniques. The findings are expected to provide theoretical support and design implications for soundscape design and optimization in smart home environments. The specific research questions include the following: (1) What indicators summarize human perception of sound (including both sound sources and the sound environment) in smart home soundscape? (2) What dimensions represent human perception of sound sources in smart home? (3) What dimensions represent human perception of the overall sound environment in smart home? Furthermore, based on the above findings, two issues were discussed: (1) the differences and relations between the perceptual dimensions of sound sources and the sound environment, and (2) the differences between the perceptual model of smart home soundscapes and existing soundscape perceptual models (the ISO and existing indoor soundscape models). The research framework is illustrated in Figure 1.

## 2 Methods

### 2.1 Qualitative research

#### 2.1.1 Interview design

Semi-structured interviews were conducted, enabling interviewees to elaborate extensively on smart home soundscape within a predetermined structural framework, while contributing supplementary information based on this framework (Acun and Yilmazer, 2018; Adeoye-Olatunde and Olenik, 2021). The interview outline comprised the following components: (1) background information (basic demographic data, such as age and place of residence, aimed at establishing a preliminary understanding of participants and facilitating follow-up inquiries), (2) sound description (interviewees' descriptions of sound sources and sound environment within the smart home), and (3) perceptual description (interviewees' subjective experiences and latent expectations concerning the described sounds). The interview guidelines are presented in Table 1.

**Table 1:** Guidelines for semi-structured interview of smart home soundscape.

Questions	Aims
1. Basic information (age, place of residence, family structure, etc.)	Demographic background
2. What smart products are used in your home? Which ones have made the strongest impression on you?	Introducing the topic
3. What kinds of sounds do the smart products in your home make? Can you describe them?	Sound source description
4. What feelings do these sounds evoke in you? What causes you to have these feelings?	Sound source perception
5. What is the sound environment like in your home? What sounds does it include?	Sound environment description
6. What feelings does this sound environment evoke in you? What causes you to have these feelings?	Sound environment perception
7. What are your expectations or suggestions regarding the sounds of smart products and the smart home soundscape?	Expectations for sound
8. Aside from this, is there anything else you would like to add?	Additional comments

#### 2.1.2 Interviewees

Participants were recruited online through social media platforms, with the criterion of being long-term smart home product users (over 2 years) who were willing to share their user experiences. The study initially recruited 30 participants, with 28 valid samples ultimately retained (average age 26.29; 10 male, 18 female). Appendix A provides detailed information on the interviewees. The average interview duration was approximately 30 min, and all interviews were audio-recorded after informed consent was obtained from the participants. During subsequent qualitative data analysis, two additional randomly selected sample datasets did not generate new coding categories, indicating that the interview sample size reached theoretical saturation.

#### 2.1.3 Qualitative coding procedure

A structured grounded theory approach was employed to code the interview data verbatim (Corbin and Strauss, 1990; Moshona et al., 2024). Grounded theory coding was adopted because it allows concepts and categories to emerge from raw data without predetermined frameworks, thereby supporting an evidence-based exploration of users' experiences. However, unlike a purely qualitative study, the aim here was not to develop a full grounded theory, but to derive indicators for the subsequent experimental quantification of the perceptual dimensions of sound sources and the sound environment. The coding protocol comprised three stages. First, open coding extracted initial sound perception attributes through line-by-line analysis of interview transcripts. Second, axial coding identifies logical connections among perceptual attributes. Finally, selective coding integrated the core perceptual categories. Data analysis was conducted using NVivo 12 software, with open coding performed independently by one researcher, and axial/selective coding was accomplished through collaborative discussions among the three researchers.

### 2.2 Quantitative research

#### 2.2.1 Sound stimuli

The sound stimuli included three components: sound source (the key experimental variable representing sounds emitted by various smart home devices), background sound (simulating baseline ambient noise in an actual smart home), and contextual sound (simulating additional environmental sounds across different experimental scenarios). Sound source stimuli were selected primarily according to the frequency of occurrence in the interview data and encompassed three types: indication, operation, and alarm sounds. Four types of sounds were chosen from the indicated sound categories: beep sound (S1), female voice (S2), male voice (S3), and musical sound (S4). The operation sound category included five types: washing machine sound (S5), robotic vacuum cleaner sound (S6), motorized curtain sound (S7), air conditioner sound (S8), and water purifier sound (S9). The alarm sound category was comprised of three types: smoke alarm sound (S10), doorbell alarm sound (S11), and air purifier alarm sound (S12), which resulted in 12 distinct sounds. Detailed classifications and explanations of sound sources are provided in Section 3.1. All the sounds were captured using a binaural recording apparatus (Head Acoustics SQobold). Sound sources, contextual sounds, and background sounds were recorded in an actual smart home living room (with no evident outdoor noise intrusion during the recording). To control the experimental variables effectively, all sound levels were calibrated to the following sound pressure levels: indication sounds at 50 dB(A), alarm sounds at 60 dB(A), operation sounds at 40 dB(A), background sounds at 35 dB(A), and contextual sounds at 50 dB(A). These sound pressure level configurations correspond to the empirical measurements of the respective sound sources (Bruck, 2001; Sato et al., 2007; BS, 2014). The spectral characteristics of each sound stimulus are presented in Appendix B. The sound stimuli were played using high-fidelity headphones (Sennheiser IE600).

#### 2.2.2 Experimental scenarios

**Figure 2:**
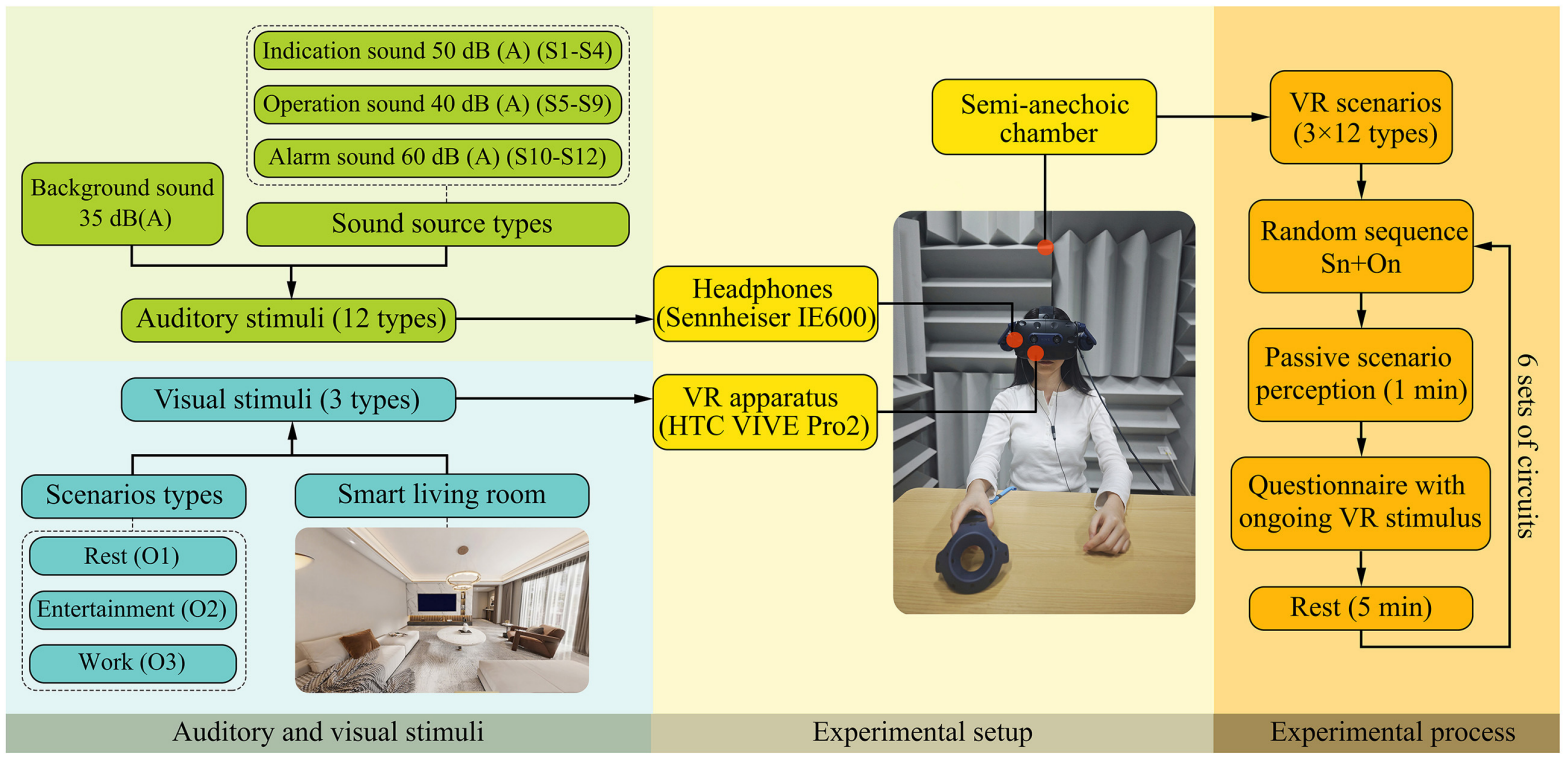
Experimental process flow diagram.

Preliminary interview data indicate that the living room represents both the longest-occupied space in residential environments and the most concentrated area for smart products. More importantly, the human activities in living rooms exhibit significant diversity. Accordingly, the experimental scenarios in this study simulated three predominant living room usage modes: entertainment, rest, and work. The spatial scale, design styles, and furnishings were referenced from frequently described configurations in interviews. The virtual environment measured 35 m^2^ with rectangular geometry, the interior design style was a modern minimalist, and there was sufficient open space to accommodate varied activity requirements (Kawai et al., 2025). The three usage modes were differentiated through television content: the rest condition (O1) was designated as the control group with the television switched off; the entertainment condition (O2) simulated viewing a film clip (playing a segment from a widely recognized ceremony film); and the work condition (O3) simulated viewing an instructional video (playing a spoken language tutorial for a less commonly taught language). VR was adopted in this study because direct experimentation in private domestic environments is difficult to standardize and replicate. The virtual living-room scene therefore served as a controlled, repeatable, and privacy-preserving platform for examining perceptual responses to smart home sounds. Accordingly, the purpose of the present experiment was to identify the perceptual structure of smart home soundscapes under controlled laboratory conditions, rather than to claim full equivalence between VR-based responses and those obtained in actual homes. Virtual environments were delivered via a VR apparatus (HTC VIVE Pro2; Hong et al., 2017; Latini et al., 2024; Ming et al., 2025), with detailed experimental scenarios provided in Figure 2.

#### 2.2.3 Questionnaire design

The questionnaire was divided into four parts: (1) 29 sound source perceptual indicators, (2) 12 sound environment perceptual indicators, (3) 8 soundscape evaluation indicators from the ISO standard (Axelsson et al., 2010; Li et al., 2024); and (4) 8 indoor soundscape evaluation indicators from a previous study (Torresin et al., 2020). The first two parts of the questionnaire were based on axial coding derived from the grounded theory analysis of the interview data (Section 3.1), while the latter two sections represented traditional and indoor soundscape evaluation methods. After merging the four duplicate evaluation indicators, the final subjective questionnaire comprised 61 evaluation terms, as shown in Table 2. All terms were rated on a 5-point Likert scale, with 1 indicating “strongly disagree” and 5 indicating “strongly agree”.

**Table 2:** Questionnaire content.

Part	Questionnaire items
Sound source evaluation (29 items)	Euphonious, Lively, Deep, Informative, Meaningful, Straightforward, Clear, Confusing, Noisy, Rackety, Cacophonous, Predictable, Sudden, Tolerable, Acceptable, Prolonged, Redundant, Distinguishable, Unique, Distinct, Shrill, Harsh, Soft, Warm, Cold, Rapid, Smooth, Novel, Interesting
Sound environment evaluation (21 items)	Orderly, Homely, Harmonized, Indistinguishable, Tolerable, Acceptable, Recognizable, Separate, Chaotic^*^, Pleasant^*^, Companionable, Belonging, Accustomed, Comfortable^*^, Confusing, Oppressive, Startling, Safe, Annoying^*^ , Irritating, Worried
ISO soundscape indicators (8 items)	Vibrant, Uneventful, Chaotic^*^, Eventful, Monotonous, Annoying^*^, Calm, Pleasant^*^
Indoor soundscape indicators (8 items)	Full of content, Private and controlled, Intrusive and uncontrolled, Empty, Engaging, Comfortable^*^, Annoying^*^, Detached

^*^Asterisks indicate duplicated indicators that overlap between the sound environment evaluation indicators and the ISO soundscape and/or indoor soundscape indicators. These duplicated indicators were merged when calculating the final set of questionnaire items.

#### 2.2.4 Participants

A total of 120 participants were recruited for the experiment (age: 18–55, M = 25.23, SD = 6.58, 50 males and 70 females). The sample size was determined by considering the experimental duration and statistical power (see Section 2.2.5). All the participants met the following inclusion criteria (Li et al., 2021): (1) prior experience using smart products, (2) self-reported normal auditory function, (3) normal corrected visual acuity, (4) absence of VR-induced cybersickness, and (5) no recent consumption of psychoactive substances. Before the experiment, all participants provided written informed consent.

#### 2.2.5 Experimental procedure

The experiment employed a factorial design with two independent variables, 12-level sound source stimuli (S1-S12) and 3-level usage scenarios (O1-O3), yielding 36 possible stimulus combinations. To mitigate fatigue effects and maintain response accuracy, each participant's experimental session was constrained to approximately 40 min. Preliminary testing determined that each participant could complete six stimulus sets, thus requiring 120 participants to yield 60 samples per sound source condition and ensuring adequate statistical power for subsequent factor analysis and ANOVA. A balanced incomplete block design was implemented to guarantee uniform frequency and sequencing of stimulus presentations across all variable levels.

The experimental sessions were conducted in a semi-anechoic chamber with an ambient noise level of 20 dB (Ba and Kang, 2019; Li and Kang, 2019). Participants received comprehensive procedural instructions prior to the experiment. Following complete comprehension, VR equipment was deployed with verbal guidance: “Please imagine yourself in your home living room while resting/entertaining/working. During this experience, you hear indications, operations, or alarm sounds from various household devices”. After acclimatization to the VR environment, the formal experiment was initiated with a randomized presentation of stimulus sets. Each sound stimulus lasted for 1 min, followed by a subjective questionnaire completion before proceeding to the subsequent stimuli until all six assigned sets were concluded. The experimental procedure is illustrated in Figure 2.

#### 2.2.6 Data analysis

Statistical analyses were performed using SPSS version 27.0. The data processing procedure involved four steps. First, the reliability and validity of the questionnaire data were tested. Second, factor analysis was used to reduce the dimensionality of perceptual indicators and to extract the potential perceptual dimensions of both sound sources and the sound environment in smart home soundscapes. Third, one-way ANOVA was conducted to examine differences among sound sources and scenarios across each perceptual dimension, with *post-hoc* comparisons performed using the Student-Newman-Keuls (S-N-K) method. Fourth, the Seaborn 0.11.2 toolkit in Python 3.8 was used for data visualization. Two-dimensional distribution plots were generated to display the spatial distribution characteristics of sound sources and scenarios derived from factor analysis. This approach reflects the variability within individual factors, including their boundary morphology and degree of dispersion, and intuitively reveals similarity relationships among different factors (Mitchell et al., 2022; Li et al., 2025). All distribution areas were represented by 50th percentile contours, enabling the comparison of distribution differences across various sound sources or scenarios within a two-dimensional subjective perceptual space.

## 3 Results

### 3.1 Indicators of smart home soundscape

This section describes how systematic grounded coding methodology can be applied to the interview data to derive the sound sources of products (AA1), sound source perceptual indicators (BB1), and sound environment perceptual indicators (CC1). Each subsection reflects the progression from initial concepts to categories, themes, and core themes. Space constraints allow only the essential coding results to be displayed.

#### 3.1.1 Product sound sources

As shown in Table 3, the sound types are divided into three main categories: indication sounds (A1), which include three subcategories that refer to proactive auditory cues emitted by products upon completing specific operations, entering particular states, or requiring user awareness of relevant information. Operational sounds (A2) comprised five subcategories representing sounds generated by internal components during normal product functioning. Alarm sounds (A3) consist of two subcategories indicating that warning sounds are activated when abnormal conditions occur or when specific alert thresholds are met. The leftmost column in Table 3 lists the sound stimuli used in the experiment, with at least one typical sound source selected from each subcategory as the experimental variable. Both male and female voices were included in the voice indication subcategory because the interview data revealed significant perceptual differences and gender preferences among users. Meanwhile, doorbell and smoke alarms were chosen within the alarm subcategory to represent the distinct secondary categories of common and uncommon alarm types, respectively.

**Table 3:** Product sound sources categories and coding framework (AA1).

The representative sounds selected for the experiment	Conceptualized data	Subcategory	Category
Beep sound (S1)	a4. Oven emits a “ding” sound	aa1. Monosyllabic indication sound	A1 indication
	a13. Washing machine emits a series of beeps after completing its cycle		
Female voice (S2) Male voice (S3)	a19. Robotic vacuum cleaner provides real-time voice updates when starting work	aa2. Voice indication sound
	a25. Floor washer provides voice indications for cleaning detection	
Musical sound (S4)	a33. Washing machine emits a melodic indication sound	aa3. Melodic indication sound
	a38. Refrigerator emits a musical sound when operational abnormalities occur	
Washing machine Sound (S5)	a81. Washing machine produces water inflow and drainage sounds during operation	aa4. Water flow sound	A2 Operation
	a51. Water purifier produces water flow sound during filtration	
Robotic vacuum cleaner Sound (S6)	a46. Robotic vacuum cleaner produces brush rotation sound during operation	aa5. Rotation sound
	a57. Washing machine produces motor operation sound during operation	
Motorized curtain sound (S7)	a41. Motorized curtains produce track sliding sound during opening and closing	aa6. Sliding sound	
Air conditioner sound (S8)	a59. Air purifier produces suction sound during filtration	aa7. Ventilation sound
	a66. Air conditioner produces blowing sound under high-speed mode	
Water purifier sound (S9)	a39. Refrigerator produces compressor operation sound during cooling	aa8. Compressor sound
	a48. Water purifier produces compressor sound during filtration	
Smoke alarm sound (S10) Doorbell alarm sound (S12)	a91. Smoke alarm sound	aa9. Personal security alarm sound	A3 Alarm
	a88. Water immersion alarm sound		
	a93. Door and window security alarm sound		
Air purifier alarm sound (S11)	a102. Air quality alarm sound	aa10. Environmental alarm sound
	a115. Air conditioner monitors abnormal environmental temperature and emits an alarm sound	

#### 3.1.2 Sound source perceptual indicators

A systematic inductive analysis of 62 initial codes for sound source perception yielded 30 mutually exclusive and saturated secondary codes (concepts) that were subsequently consolidated into 14 tertiary codes (categories) and 29 subcategories (Table 4). All categories and subcategories were rigorously grounded in the users' original expressions, maintaining linguistic authenticity while achieving conceptual abstraction. Notably, certain categories encompass multiple semantically proximate subcategories (e.g., B1 contains both Euphonious and Lively). Given the substantial semantic overlap among subcategories and to prevent subjective aggregation bias, categorical nomenclature was deliberately preserved at the subcategory level during the qualitative analysis. To mitigate individual interpretation variances in the questionnaire responses, the indicators directly employed subcategory-level descriptors as measurement items (e.g., maintaining B1a Euphonious and B1b Lively as distinct questionnaire items). The ultimate validation and denomination of the core categories were performed through subsequent quantitative analysis.

**Table 4:** Sound source perceptual categories and coding framework (BB1).

Example of excerpts from the interview (original descriptions)	Subcategory		Category
“The refrigerator's indication sound is quite euphonious – moderate in volume, and with a pleasant melody”.	B1a	Euphonious	B1
“The washing machine's completion sound is very lively. It plays a ‘ding-ding-ding' melody every time it finishes a cycle”.	B1b	Lively	
“At night, you can hear a deep, humming water-like sound from the air purifier”.	B2a	Deep	B2
“This sound provides a better indication that it is working. Without it... I wouldn't know whether it is operating”.	B3a	Informative	B3
“Through voice indications, I can tell which step it is in, and I can make judgments based on the indications...”	B3b	Meaningful	
“The floor washer's voice indications are indeed quite accurate – for example, ‘The wastewater tank is full' or ‘Please replace the clean water tank'...”	B4a	Straightforward	B4
“Indications like ‘Start cleaning' are very clear to me”.	B4b	Clear	
“The washing machine's indication is just a simple beep sound, which sometimes leaves me uncertain about its meaning”.	B4c	Confusing	
“When the range hood operates at high speed, it is really noisy. Sometimes... I can't even hear anything around me”.	B5a	Noisy	B5
“If the robotic vacuum cleaner starts working early on weekends, I find it too rackety”.	B5b	Rackety	
When the washing machine's motor runs, it hits the surrounding walls, and the sound of the drum spinning is very cacophonous'.	B5c	Cacophonous	
“The indication sound that each appliance makes when it is completed has a certain psychological expectation for me”.	B6a	Predictable	B6
“If I forget to clean the water tank, it suddenly emits an alarm sound when it is time for regular cleaning”.	B6b	Sudden	
The dishwasher makes noise when in use, but... personally, I find it within my tolerance level'.	B7a	Tolerable	B7
“When the water purifier is filtering water... I think the sound is actually acceptable”.	B7b	Acceptable	
The soymilk maker... keeps beeping incessantly if you don't open the lid after it finishes'.	B8a	Prolonged	B8
When I use voice commands to control it... it starts playing the startup sound, which feels a bit redundant'.	B8b	Redundant	
“Even for products from the same brand, their sounds have some differences... Over time, you can learn to distinguish them”.	B9a	Distinguishable	B9
“My rice cooker doesn't make the usual ‘beep beep' sound—it produces a single ‘ding,' which is very clear”.	B9b	Unique	
“From the sound of the air purifier's fan, I can tell whether it is on high mode or silent mode”.	B9c	Distinct	
“The sound of my washing machine after finishing laundry is so shrill that it can be heard from three floors away”.	B10a	Shrill	B10
“When the vacuum cleaner is operating... the sound is still slightly harsh to the ears”.	B10b	Harsh	
“The sound of the water purifier filtering is very soft. It is unobtrusive... a very soft sound”.	B11a	Soft	B11
“I find Xiao Ai's voice quite warm and gentle”.	B12a	Warm	B12
“I think the sound of this robotic vacuum cleaner is the coldest one in our house”.	B12b	Cold	
“When the robot vacuum gets stuck on something, the sound it makes feels too rapid”.	B13a	Rapid	B13
“When it sprays mist outside with water, the sound is smooth and soft. I find it quite pleasant”.	B13b	Smooth	
“My refrigerator's indication sound is a short song—it feels fresh and quite innovative”.	B14a	Novel	B14
“Sometimes when the robotic vacuum cleaner turns a corner or avoids an obstacle, it makes a sound—it's really interesting”.	B14b	Interesting	

#### 3.1.3 Sound environment perceptual indicators

The analysis yielded 58 initial codes for sound environment perception, which were refined into 32 secondary codes and subsequently consolidated into 16 categorical dimensions (C1-C16) comprising 21 subcategories (Table 5). These dimensions collectively capture the users' holistic subjective experiences with smart home soundscapes. Comparative analysis between Tables 4, 5 reveals three fundamental distinctions between sound source and sound environment perceptions in smart home contexts. First, the descriptive focus differs: sound source perception targets the sounds themselves, whereas sound environment perception emphasizes integrative experiential qualities. Second, sound environment perception is more closely connected with personal affective experiences and therefore shows stronger dependence on specific scenarios and activities. Third, this heightened contextual sensitivity enables sound environment perceptual indicators to exhibit more pronounced temporal and spatial characteristics than sound source perceptual indicators.

**Table 5:** Sound environment perceptual categories and coding framework (CC1).

Example of excerpts from the interview (original descriptions)	Subcategory		Category
“They operate without interfering with each other; each takes its turn to emit sounds in sequence. I find this quite good”.	C1a	Orderly	C1
“When they do not affect people's normal lives, the sounds of these appliances are harmonious and gives the household a homely feeling”.	C2a	Homely	C2
“Their operational humming blends into the background, forming a kind of base noise in the home”.	C3a	Harmonized	C3
“If the television or game audio is too loud, I cannot hear the washing machine's completion sound”.	C3b	Indistinguishable	
“The sounds produced by various smart products operating in the home environment are generally within an acceptable range and do not cause noticeable interference with daily life”.	C4a	Tolerable	C4
“The overall sound environment currently remains within acceptable limits”.	C4b	Acceptable	
Even amidst the blended sounds of a television and other smart devices, the air fryer's roar remains recognisable'.	C5a	Recognizable	C5
“Their sounds seem to occupy two different channels, resulting in an impression of clearly separate layers”.	C5b	Separate	
“Simultaneous activation of the refrigerator's abnormal alarm and television sound creates a sense of auditory chaos”.	C6a	Chaotic	C6
“The orderly operation sounds of smart home appliances create a pleasantly harmonious atmosphere”.	C7a	Pleasant	C7
“Sometimes the sounds from these appliances give me the comforting sense of having companionship at home”.	C8a	Companionable	C8
“The accompanying sounds produced by these appliances while they operate are like a companion, giving me a strong sense of belonging”.	C8b	Belonging	
“Under the circumstances, I feel quite accustomed to the overall auditory environment”.	C9a	Accustomed	C9
“The sound of the smart devices operating at home is a deep, steady sound, not complete silence, creating a comfortable atmosphere”.	C10a	Comfortable	C10
“The similarity in the alert tones of several appliances creates confusion; when operating together, it's difficult to identify them”.	C11a	Confusing	C11
“When I was watching TV, the robotic vacuum cleaner malfunctioned, and its sound was persistent and repetitive. I immediately handled it because at that moment, the overall sound environment gave me a sense of pressure”.	C12a	Oppressive	C12
“When I am focused on other things, the smart speaker suddenly starts reporting the weather; this unexpected sound intrusion stands out particularly in the overall home soundscape and can startle me”.	C13a	Startling	C13
“When the sound environment is quiet, the video doorbell scans and indicates if someone lingers at the door, keeping me informed and making me feel safe”.	C14a	Safe	C14
“No matter how expensive or advanced the air purifier is, in the quiet sound environment of nights, you can still find it a little annoying”.	C15a	Annoying	C15
“That sound lasted a bit too long, and no matter what I was doing at the time, I had to stop to deal with it because the chaotic sound environment at that moment made me very irritated”.	C15b	Irritating	
“Sometimes, when using hot water, the gas water heater suddenly starts to make a beep sound. This sharp noise directly disrupts the harmony of the sound environment, causing a lot of worry and tension”.	C16a	Worried	C16

### 3.2 Perceptual dimensions of sound sources

#### 3.2.1 Construction of sound source perceptual dimensions

**Figure 3:**
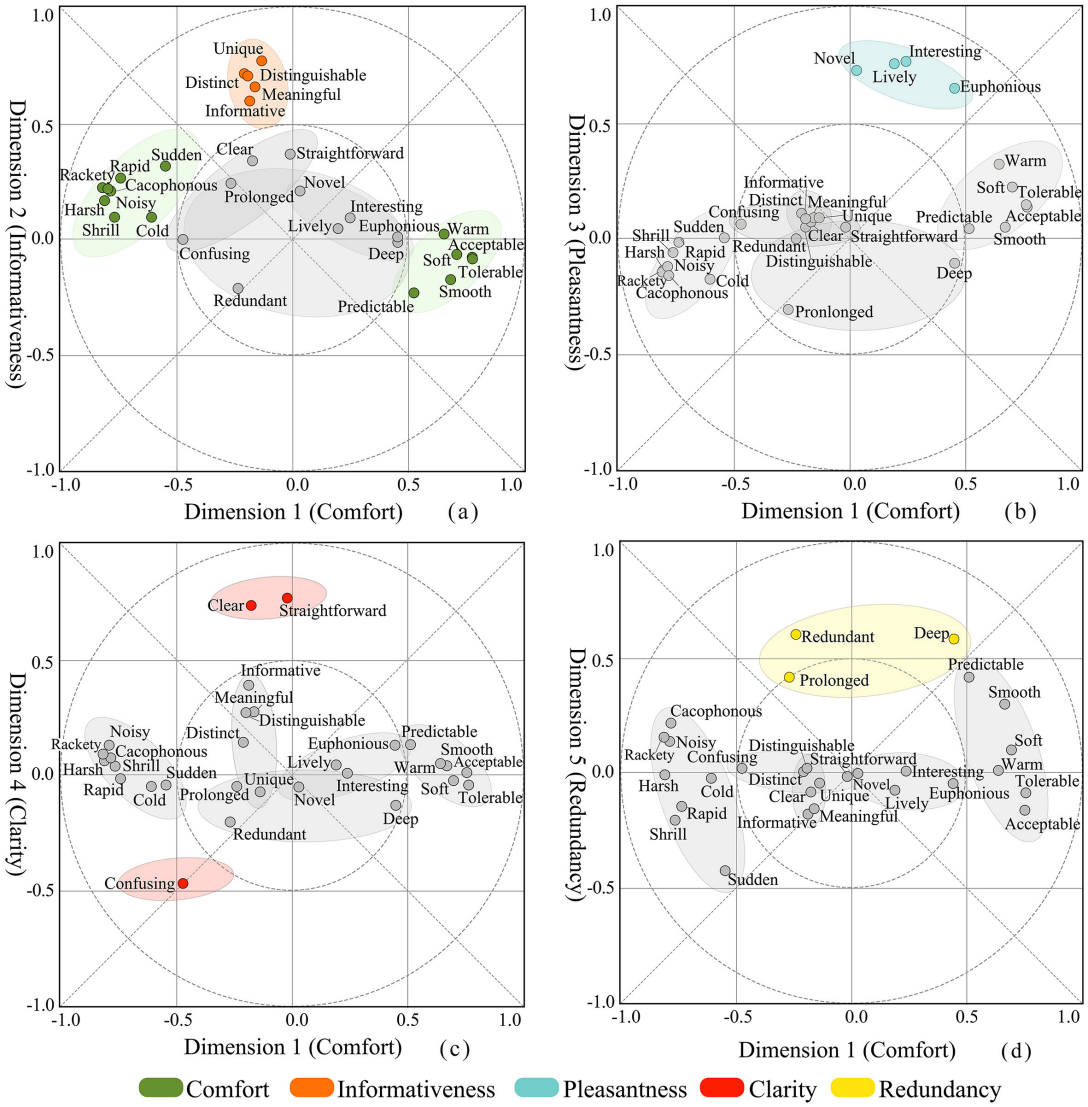
Factor loading plot of sound source perceptual dimensions: **(a)** Dimension 1 (Comfort) and Dimension 2 (Informativeness); **(b)** Dimension 1 (Comfort) and Dimension 3 (Pleasantness); **(c)** Dimension 1 (Comfort) and Dimension 4 (Clarity); and **(d)** Dimension 1 (Comfort) and Dimension 5 (Redundancy).

Questionnaire-derived data demonstrated satisfactory reliability and validity: overall reliability (Cronbach's α) reached 0.880, with excellent construct validity across variables (KMO=0.923). During the factor analysis procedure, orthogonal rotation was employed to extract common factors, and varimax rotation was applied to achieve an explainable structure. Following the eigenvalue-greater-than-1 criterion, five main dimensions were extracted from the sound source perceptual indicators (the rotated factor loadings are detailed in Appendix C). For enhanced visualization, spatial loading plots were generated using Dimension 1 as the reference (Figure 3).

Figure 3a reveals that Dimension1′s positive loading comprised warm, soft, acceptable, smooth, tolerable, and predictable, whereas the negative pole included noisy, rackety, harsh, shrill, sudden, rapid, cacophonous, and cold. These indicators can be summarized as the Comfort dimension. Dimension2′s positive loadings (unique, distinct, distinguishable, meaningful, and informative) indicate an Informativeness dimension. Figure 3b shows that Dimension3′s positive loading (euphonious, lively, interesting, and novel) constitutes the Pleasantness dimension. Figure 3c demonstrates Dimension4′s positive loading (straightforward and clear) and negative loading (confusing) form a Clarity dimension. Figure 3d reveals that Dimension5′s positive loading is derived from redundant, deep, and prolonged, although with limited contributions (all < 0.65) and weak semantic coherence. Predictable and sudden provide supplementary positive and negative contributions, respectively. This indicates that the structure of Dimension 5 lacks cohesion, with insufficient logical consistency among the indicators and can only be tentatively summarized as a Redundancy dimension.

Factor analysis yielded five main perceptual dimensions of sound sources in smart home soundscapes: Comfort, Informativeness, Pleasantness, Clarity, and Redundancy. While Comfort and Pleasantness demonstrate semantic proximity, they constitute separate dimensions: Comfort predominantly characterizes the acceptability of product sounds, whereas Pleasantness primarily denotes the novelty of sound events. In other words, in certain extreme cases, sounds may elicit pleasant (or excitatory) responses despite being uncomfortable. Consequently, the Pleasantness dimension exhibits higher subjectivity and transcends conventional comfort. Similarly, Informativeness and Clarity share conceptual similarities but differ functionally: Informativeness operates at a foundational level, reflecting mere signal reception, while Clarity engages in higher cognitive processing, indicating the comprehensibility of auditory information. Thus, these two dimensions represent the successive phases of auditory cognition.

#### 3.2.2 Distribution of sound source perceptual dimensions

**Figure 4:**
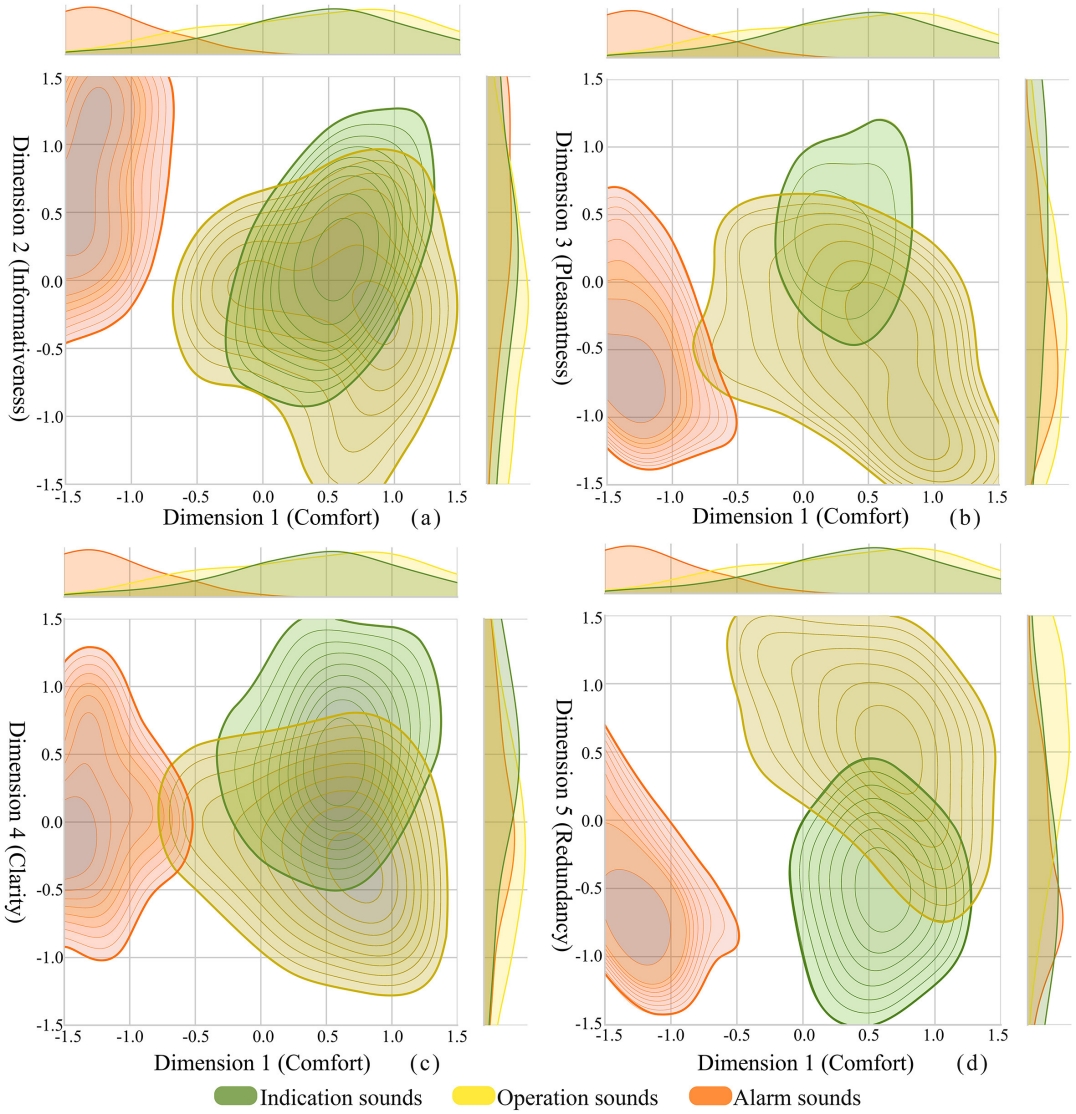
Principal component distribution of sound source perception for three sound source types: **(a)** Dimension 1 (Comfort) and Dimension 2 (Informativeness); **(b)** Dimension 1 (Comfort) and Dimension 3 (Pleasantness); **(c)** Dimension 1 (Comfort) and Dimension 4 (Clarity); and **(d)** Dimension 1 (Comfort) and Dimension 5 (Redundancy).

Building on the five identified perceptual dimensions of sound sources, an ANOVA was conducted to examine whether significant differences existed across different types of sound sources along these perceptual dimensions. *Post hoc* analysis was performed using the S-N-K method. The simplified results are presented in Table 6, where homogeneous subsets are indicated by uppercase letters. All statistical tests used a 95% confidence interval. Figure 4 shows the distribution of the principal components of the three types of sound source.

**Table 6:** ANOVA and *post-hoc* test results for sound source and scenario types across sound source perceptual dimensions.

Independent variable						
Sound source type	Dimension	F	p	Indication	Operation	Alarm
	Dimension 1 (Comfort)	401.597	< 0.001	0.413 (B)	0.424 (B)	−1.258 (A)
	Dimension 2 (Informativeness)	38.080	< 0.001	−0.010 (B)	−0.289 (A)	0.495 (C)
	Dimension 3 (Pleasantness)	61.642	< 0.001	0.541 (B)	−0.258 (A)	−0.292 (A)
	Dimension 4 (Clarity)	28.254	< 0.001	0.352 (C)	−0.275 (A)	−0.011 (B)
	Dimension 5 (Redundancy)	130.659	< 0.001	−0.486 (A)	0.609 (B)	−0.366 (A)
Scenario type	Dimension	F	p	Rest	Entertainment	Work
	Dimension 1 (Comfort)	2.190	0.113	−0.110 (A)	0.062 (A)	0.048 (A)
	Dimension 2 (Informativeness)	6.747	0.001	−0.061 (A)	−0.127 (A)	0.188 (B)
	Dimension 3 (Pleasantness)	0.450	0.638	−0.045 (A)	0.041 (A)	0.004 (A)
	Dimension 4 (Clarity)	3.137	0.044	0.131 (A)	−0.059 (A)	−0.072 (A)
	Dimension 5 (Redundancy)	0.200	0.819	0.030 (A)	−0.027 (A)	−0.003 (A)

Table 6 combined with Figure 4 reveal differences in sound source perception. Figure 4a demonstrates that indication and operation sounds predominantly occupy Dimension1′s positive domain, whereas alarm sounds cluster in the negative region with significant differentiation, indicating substantially reduced comfort for alarms. Although no significant difference was observed between indication sounds and operation sounds on Dimension 1, the latter were associated with marginally higher perceived comfort. This finding is potentially attributable to their lower sound pressure levels and greater inter-individual variability in preference for operational sounds. Dimension 2 displays alarm sounds in the positive domain, indication sounds clustered around the center, and operation sounds in negative domain, all pairwise comparisons reaching significance. This indicates maximal informativeness for alarm sounds and minimal for operation sounds. Figure 4b reveals indication sounds dominating Dimension3′s positive region, significantly exceeding alarm and operation sounds in the negative domain, suggesting superior pleasantness for indication sounds. Figure 4c shows indication sounds in Dimension4′s positive domain, alarm sounds around centered, and operation sounds in negative domain, all differing significantly. This further validates the distinction between Dimension 2 (Informativeness) and Dimension 4 (Clarity)—A potential explanation may be that alarm sounds occur less frequently in daily contexts, thereby eliciting heightened vigilance among listeners. This increased alertness consequently enhances the perceived informativeness of such signals. In contrast, indication sounds are more commonplace and often require subjective interpretation of their meaning. This necessity for cognitive engagement may account for their higher ratings on Dimension 4 (Clarity). Figure 4d indicates operation sounds occupying Dimension5′s positive domain, significantly diverging from alarm and indication sounds in the negative domain, implying strongest redundancy perception for operation sounds.

**Figure 5:**
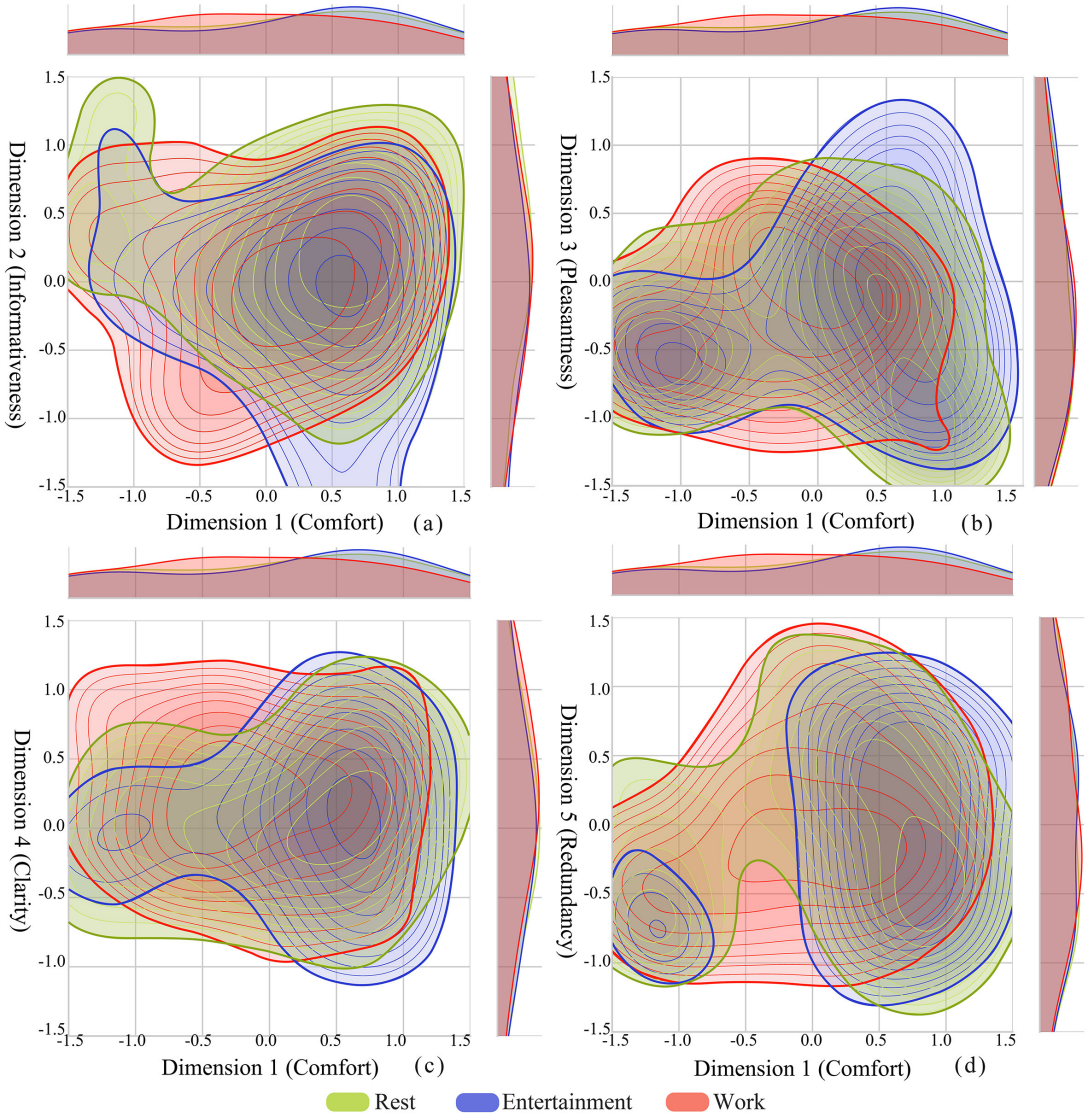
Principal component distribution of sound source perception for three scenario types: **(a)** Dimension 1 (Comfort) and Dimension 2 (Informativeness); **(b)** Dimension 1 (Comfort) and Dimension 3 (Pleasantness); **(c)** Dimension 1 (Comfort) and Dimension 4 (Clarity); and **(d)** Dimension 1 (Comfort) and Dimension 5 (Redundancy).

ANOVA with a *post-hoc* test was used to examine sound source perception differences among scenario types (Table 6), with the corresponding distributions illustrated in Figure 5. Table 6 demonstrates non-significant scenario effects across all perceptual dimensions, except for Dimension 2. Figure 5 confirms this pattern through a substantial distributional overlap across scenarios. Regarding Dimension 2, the rest of the scenarios exhibited significantly higher ratings than the work and entertainment scenarios, although this differentiation remains visually subtle in Figure 5a, indicating a marginally enhanced informativeness evaluation during resting states. A comparative analysis of Table 6 alongside Figures 4, 5 establishes that scenario types exert markedly weaker effects on sound source perception than sound source types.

### 3.3 Sound environment perceptual dimensions

#### 3.3.1 Construction of sound environment perceptual dimensions

**Figure 6:**
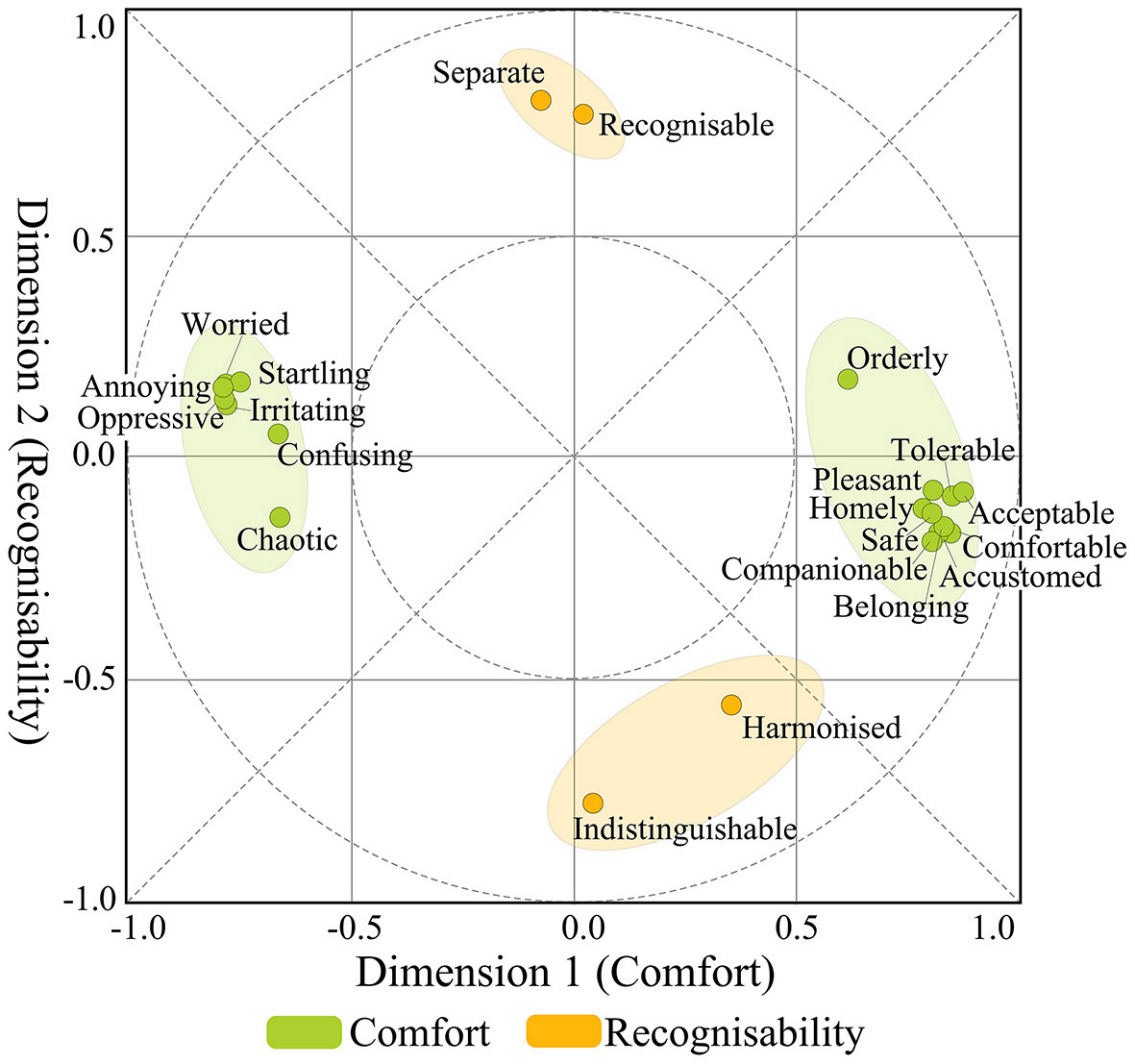
Factor loading plot of sound environment perceptual dimensions.

The questionnaire for sound environment perception demonstrated satisfactory overall reliability (Cronbach's alpha = 0.923), and the construct validity of all variables was excellent (KMO = 0.951). Factor analysis was conducted on the 21 sound environment evaluation indicators using the same parameter settings as Section 3.2.1. Ultimately, two perceptual dimensions of the sound environment were identified. The factor loadings are detailed in Appendix D, and a two-dimensional factor-loading plot is presented in Figure 6.

Figure 6 reveals that Dimension1′s positive loading comprises ten variables: comfortable, tolerable, belonging, accustomed, companionable, safe, pleasant, homely, orderly, and acceptable, whereas the negative loading contains seven variables: oppressive, annoying, irritating, worried, confusing, startling, noisy, and chaotic. Thus, this dimension is conceptualized as Comfort dimension. Dimension2′s positive loading features are separate and indistinguishable, while its negative loading are recognizable and harmonized, leading to its interpretation as Recognisability dimension.

#### 3.3.2 Distribution of sound environment perceptual dimensions

**Figure 7:**
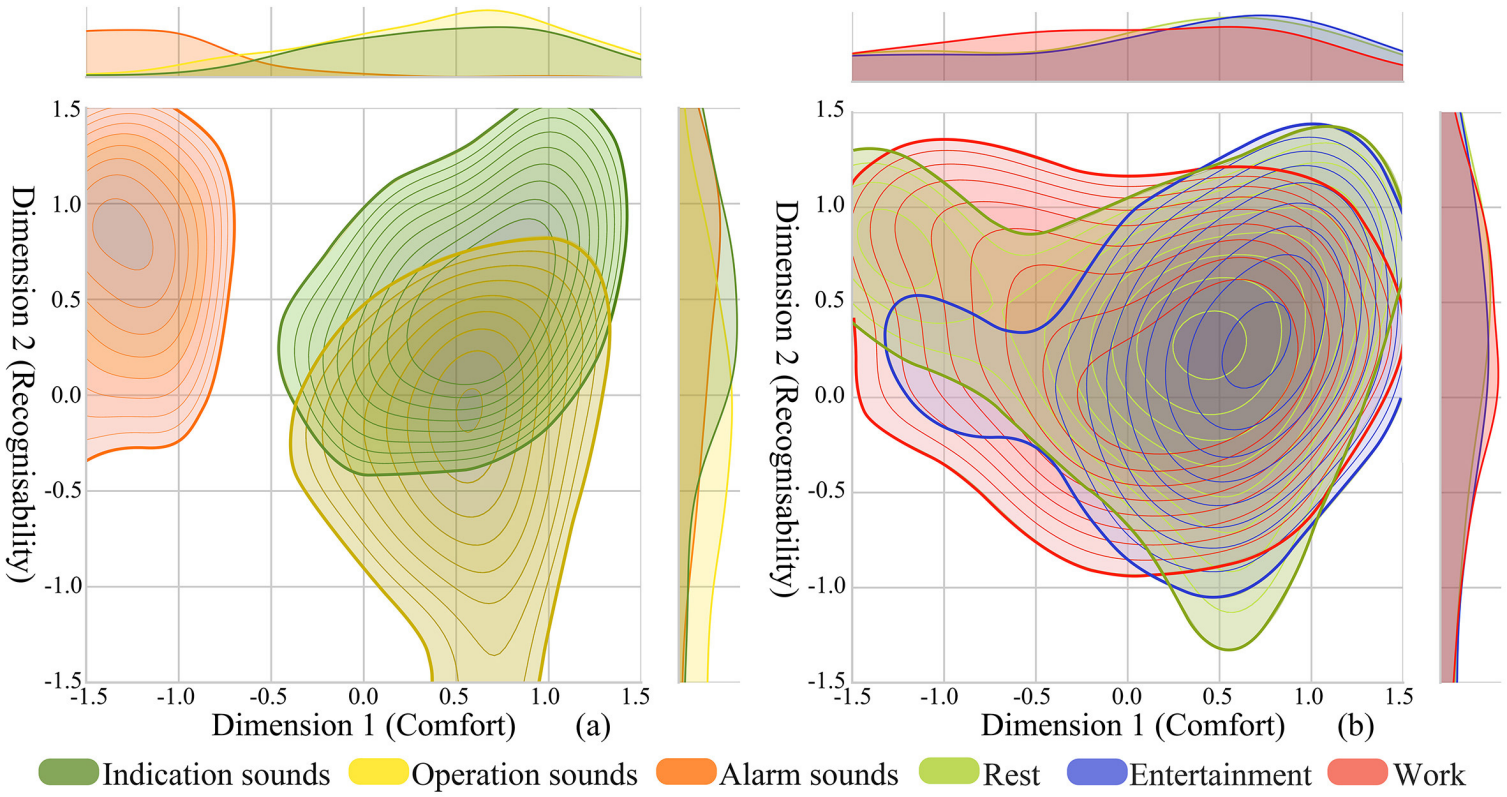
Principal component distribution of sound environment perception: **(a)** distributions across three sound source types; and **(b)** distributions across three scenario types.

ANOVA was employed to investigate the effects of sound source and scenario type on perceptual dimensions, with the S-N-K method used to calculate homogeneous subsets. The results are presented in Table 7 and the corresponding distribution plots are shown in Figure 7. Because the sound environment perception comprises only two dimensions, the results for the sound source and scenario types are discussed collectively.

**Table 7:** ANOVA and *post-hoc* test results for sound source and scenario types across sound environment perceptual dimension.

Independent variable						
Sound source type	Dimension	*F*	*P*	Indication	Operation	Alarm
	Dimension 1 (Comfort)	465.737	< 0.001	0.458 (B)	0.414 (B)	−1.301 (A)
	Dimension 2 (Recognisability)	76.289	< 0.001	0.335 (B)	−0.495 (A)	0.379 (B)
Scenario type	Dimension	F	p	Rest	Entertainment	Work
	Dimension 1 (Comfort)	3.803	0.023	−0.140 (A)	0.101 (B)	0.040 (B)
	Dimension 2 (Recognisability)	0.877	0.416	0.060 (A)	−0.060 (A)	−0.001 (A)

Figure 7a shows that indication and operation sounds occupy the positive domain of Dimension 1, whereas alarm sounds cluster in the negative domain with a statistically significant differentiation, demonstrating that alarm sounds substantially diminish acoustic comfort. Operation sounds are distributed in Dimension2′s positive domain, significantly diverging from indication and alarm sounds in the negative domain. This may be due to the long-term presence and relative stability of operation sounds in domestic settings, yielding high integration perception, or possibly to their lower sound pressure levels and rhythmic characteristics.

Figure 7b shows substantial distributional overlap among the three scenario types, indicating limited scenario-based influence on sound environment perception. Integrating Table 7 data, only work scenario registers significantly lower Dimension 1 scores than other scenarios, reflecting heightened comfort demands during work activities. All scenarios demonstrate non-significant differences on Dimension 2 with widely scattered data (means approximating zero), confirming scenario factors' negligible impact on recognisability perception.

## 4 Discussion

### 4.1 Differences and relations between sound sources and sound environment in perceptual dimensions

**Figure 8:**
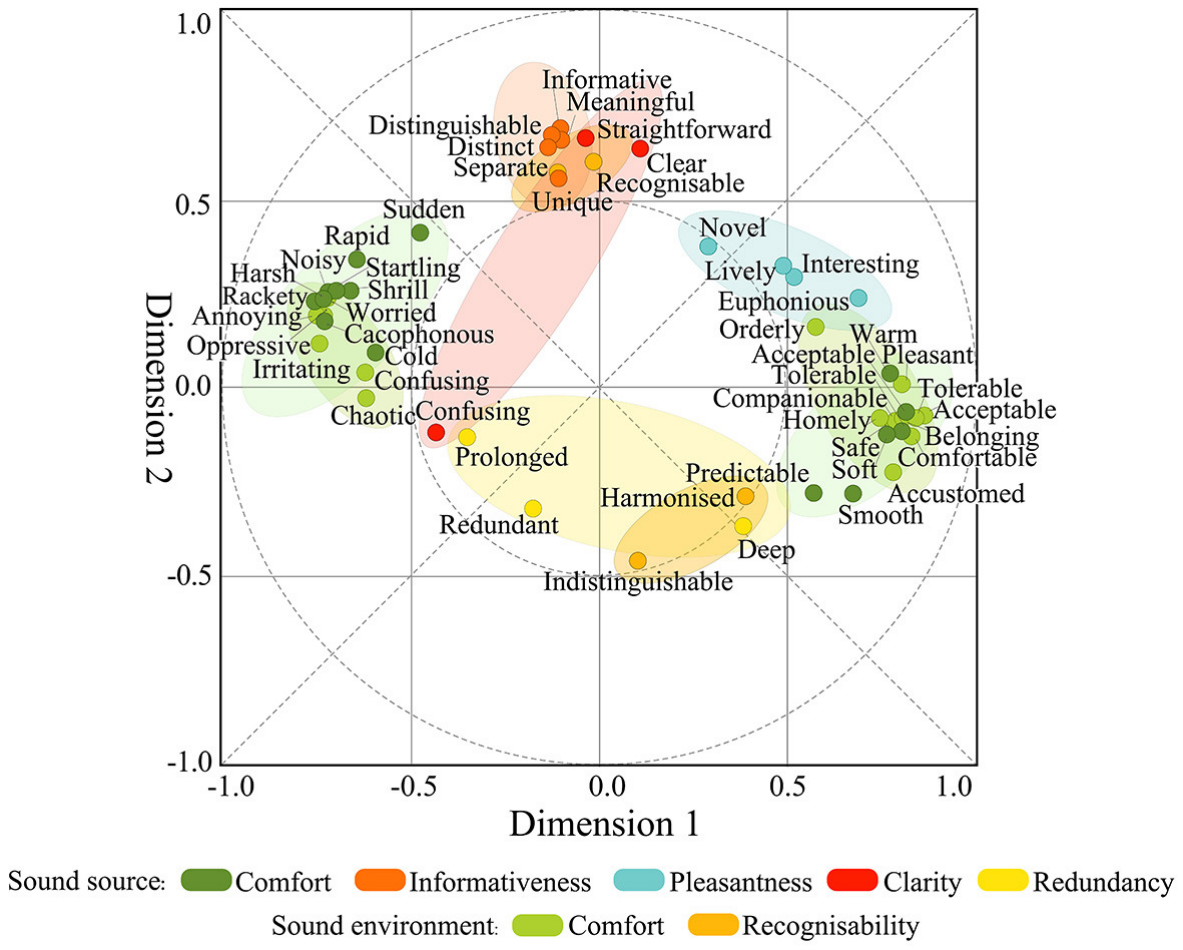
Factor loading plot of sound source and sound environment perceptual dimensions.

The factor analysis of 29 sound source perceptual indicators and 21 sound environment perceptual indicators produced a two-dimensional factor loading plot, as shown in Figure 8. Notably, this section focuses on examining perceptual dimension differences and relations between the sound source and the sound environment, not deriving precise common factors. Despite the results suggesting more than two factors, Figure 8 effectively visualizes the interfactor relationships through an optimal two-dimensional observational framework.

The synthesized findings from Sections 3.2 and 3.3 reveal the dimensional relations between sound source and sound environment perceptions. Both share Dimension 1 (Comfort), establishing it as the most fundamental and critical requirement for smart home soundscapes. Dimension 2 (Informativeness) and Dimension 4 (Clarity) of sound source demonstrate substantial convergence with the sound environment's Dimension 2 (Recognisability), indicating sound information recognition as the secondary essential requirement. Differences also exist: sound source perception Dimension 3 (Pleasantness) is distributed diagonally across Figure 8′s two-dimensional plane, suggesting that it operates simultaneously on both sound environment perceptual dimensions rather than primarily aligning with either. Thus, enhanced Pleasantness may concurrently improve Comfort and Recognisability. Conversely, sound source perception's Dimension 5 (Redundancy) exhibits limited contribution (most loadings < 0.5), confirming its conceptual ambiguity and minimal influence on sound environment perception.

These findings demonstrate that the progression from sound source to sound environment perception constitutes a complex cognitive process. As such, this study can only provide a preliminary qualitative discussion of their relationship, precluding quantitative causal characterization. Actual causality likely involves bidirectional interactions. While sound source perception influences environmental perception (Joynt and Kang, 2010; Lian et al., 2025), sound environment perception may reciprocally affect sound source interpretation (Calcagno et al., 2012; Spiousas et al., 2017). These interactions may intensify with smart-device integration. Subsequent investigations should include a broader variety of sound sources. More advanced statistical approaches, such as multivariate linear regression (Deng et al., 2015; Uebel et al., 2025) and structural equation modeling (Hong and Jeon, 2015; Tarlao et al., 2021), should also be adopted to quantify the relationship between sound sources and sound environment perceptions in smart homes.

### 4.2 Comparison with established soundscape perception models

**Figure 9:**
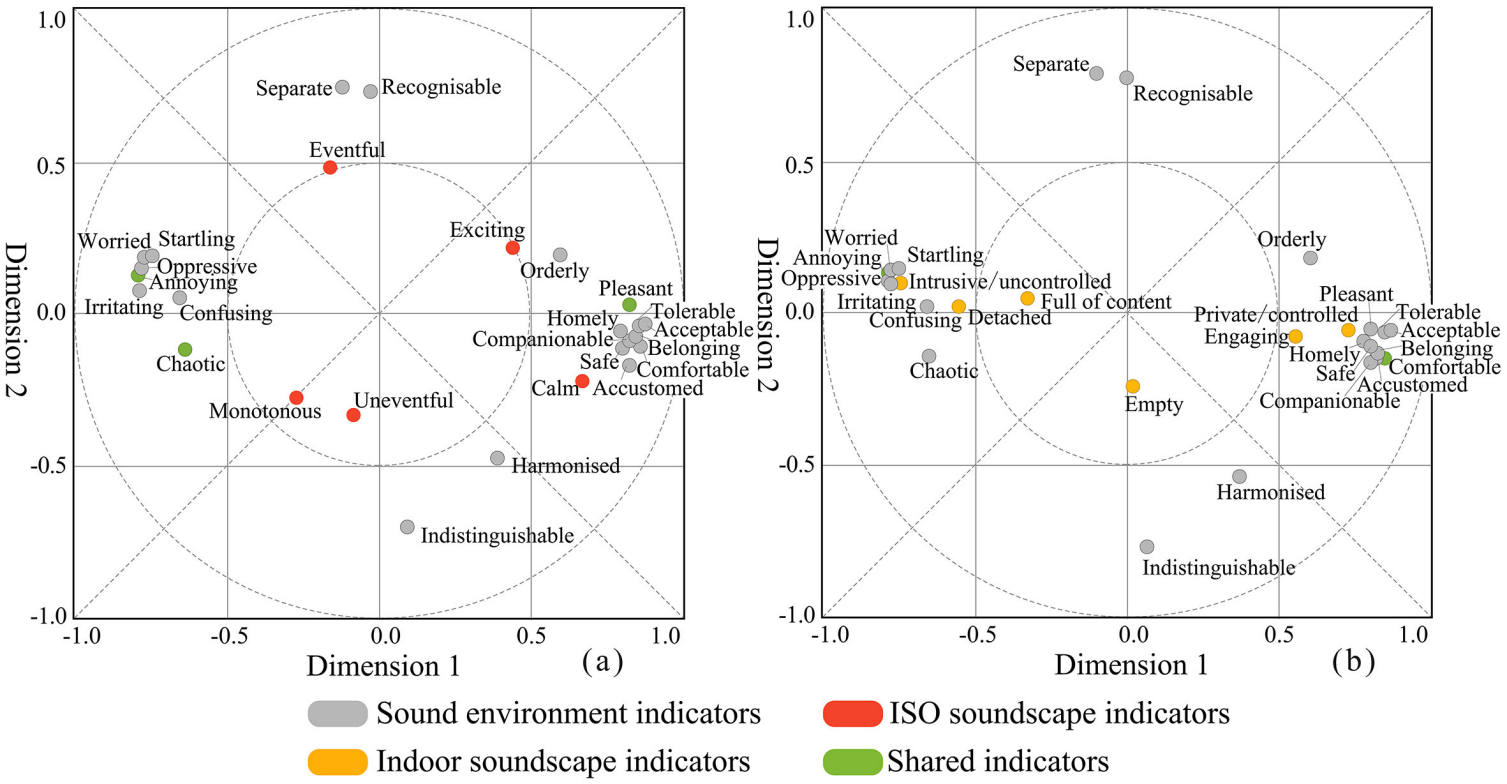
Factor loading plot comparing the smart home soundscape model with existing soundscape models: **(a)** comparison with ISO soundscape indicators; and **(b)** comparison with indoor soundscape indicators.

Factor analysis was conducted jointly on 21 sound environment perceptual indicators and eight ISO soundscape evaluation indicators, generating a two-dimensional factor loading plot, as shown in Figure 9a. It can be observed that Dimension 1 of smart home sound environment is coaxial with Dimension 1 of ISO soundscape, both representing the Comfort/Pleasantness dimension. Meanwhile, ISO's Dimension 2 (Uneventful-Eventful) largely overlaps with Dimension 2 (Recognisability) of smart home sound environment, indicating that Eventful is positively correlated with Recognisability, while Uneventful is positively correlated with Unrecognisability. However, most ISO soundscape indicators have loadings below 0.5, suggesting that the smart sound environment perceptual model is more suitable than the ISO perceptual model for evaluating the indoor soundscape quality in smart buildings. This overlap indicates partial convergence between the two models, but does not imply conceptual equivalence. In the ISO framework, Eventfulness mainly reflects the perceived level of sonic activity or environmental richness, whereas Recognisability in smart homes places greater emphasis on whether automated and device-related sounds can be clearly identified, semantically interpreted, and integrated into everyday domestic routines. Therefore, Recognisability is not merely another expression of the traditional valence-arousal structure, but reflects a more functional and cognitive characteristic of smart home soundscape perception. Additionally, the results imply that the outdoor sound environment perception emphasizes richness and diversity, whereas the sound environment perception of smart homes focuses more on informativeness and recognisability.

Similarly, a factor analysis was performed on the sound environment indicators of smart homes and eight indoor soundscape evaluation indicators (Figure 9b), which shows that Dimension 1 of the indoor soundscape is also coaxial with Dimension 1 of smart home sound environment perception. This indicates that regardless of indoor or outdoor contexts or the introduction of smart home concepts, Pleasantness and Comfort remain the most important dimensions of sound environment perception. However, in Dimension 2, the indoor soundscape model collapsed more completely than the ISO model, with the full content having almost zero loading on Dimension 2. Two reasons may explain this. First, the actual acoustic condition in a smart home soundscape inherently involves numerous sound events, with indication sounds occurring unexpectedly. In other words, smart home soundscapes are inherently full of content and uncontrolled, and users who choose to use smart home systems and live within them may already have been psychologically prepared or adapted to such complex sound environments. Thus, the evaluation dimensions of the full content may have exceeded the threshold and become difficult to measure. Second, this study did not introduce outdoor sound sources (no interviewees mentioned interference from outdoor sounds), which differs from the settings of Torresin et al. (2020). Therefore, many intrusive outdoor noises may affect indoor sound environment perception; however, they were not included in the current model as this study focused exclusively on indoor sound sources. Taken together, these comparative findings offer design implications for smart home soundscape design, particularly in relation to balancing comfort, informativeness, and recognisability in automated sound design.

### 4.3 Limitation and future studies

The first limitation pertains to laboratory constraints, ecological validity, and the contextual boundary of the simulated setting. This study captured only short-term user perceptions of smart home soundscapes, whereas long-term adaptation effects remained unmeasurable through experiments. Although the setup was informed by interview data to construct a representative smart home space deemed realistic by most participants, perceptual responses in a VR-based environment may still differ from those in actual domestic settings. In the present study, VR was adopted as a controlled, repeatable, and privacy-preserving platform for examining smart home soundscape perception, rather than as a substitute for *in situ* residential experience. Individual variability in environmental adaptation capacity (Baker and Fairclough, 2022; Versümer et al., 2023) and potential disparities between short-term evaluations and long-term product adaptability (Aletta et al., 2018) may therefore influence smart home environment perceptions, warranting future validation. In addition, the simulated scenarios were primarily differentiated through television content and did not incorporate more explicit task-driven activities or behavioral interactions, while the virtual environment was fixed to a relatively orderly modern minimalist interior. These controlled settings may have constrained contextual variation, and users may respond differently under other interior styles, levels of household organization, or broader multisensory domestic contexts.

Second, while the sound stimuli represented the most prevalent sound sources in the interviews, the study excluded combined sound source occurrences. As discussed in Section 4.1, real-world smart home soundscapes can exhibit greater complexities. Consequently, this phase of research maintained an analytical separation between sound source and sound environment perceptions. The interrelationship between these perceptual layers in smart home contexts, along with the combinatorial effects of sound sources on sound environment perception, require further exploration.

Finally, all the participants in this study were familiar with smart home environments and had experience using smart devices. Although they represent the primary user base for smart homes, just as tech opportunists tend to have higher product requirements (Venesz et al., 2022), users who actively pursue smart lifestyles may perceive sound environments differently than those who passively live in smart homes. Additionally, cultural background differences may influence sound perception, as has been extensively demonstrated in outdoor soundscape research (Mohamed and Dokmeci Yorukoglu, 2020; Yang et al., 2024). All these factors could potentially affect the perceptual model of smart home sound environments.

In summary, to refine the existing model and improve its ability to explain more phenomena, future research could focus on the following three aspects: (1) combining field studies and actual measurements to compare differences between perceptions of real smart home soundscapes and laboratory conditions; (2) introducing more sound sources to simulate composite sound conditions and employing more diverse statistical methods to explore quantitative relationships between sound sources and sound environment perceptions in smart homes; and (3) investigating differences among user groups with varying levels of smart product acceptance in their perception of the smart home soundscape.

## 5 Conclusions

This study employed a mixed-methods framework, this research leveraged grounded theory and VR-based experiments to develop a perceptual model of the smart home soundscape, identifying its core dimensions and influential factors. The main conclusions are as follows:

(1) Sound sources in smart homes were categorized into three types (indication, operation, and alarm sounds), sound source perception was summarized into 14 categories (29 evaluation indicators, such as Euphonious, Noisy, and Informative), and sound environment perception was summarized into 16 categories (21 evaluation indicators, such as Comfortable, Chaotic, and Companionable).

(2) Five dimensions were extracted for sound source perception in smart homes (Comfort, Informativeness, Pleasantness, Clarity, and Redundancy). Among these, Comfort emerged as the most fundamental and critical dimension. Pleasantness and Clarity demonstrated higher subjectivity and involved more advanced cognitive processing compared to Informativeness, while Redundancy constituted a conceptually weaker factor. These dimensions demonstrated distinct patterns across sound types, with alarms being informative but uncomfortable, indications pleasant and clear, and operations comfortable yet redundant. Scenario influence was minimal.

(3) Two dimensions were extracted for the sound environment perception in smart homes (Comfort and Recognisability). These two dimensions correspond to the affective level (Comfort) and the cognitive level (Recognisability), respectively, which together form a comprehensive assessment of the sound environment. Overall, sound environment perception was predominantly influenced by the type of sound source rather than scenario type (rest, entertainment, and work).

(4) Sound environment perception emerges from sound sources through a complex cognitive process. Comfort is the primary shared dimension, followed by the convergence of sound information dimensions (Informativeness, Clarity, Recognisability) as a secondary requirement. Pleasantness uniquely influences both levels, unlike the minimal role of Redundancy.

(5) Compared with established models, the smart home soundscape model shows both convergence and specificity. Its Comfort dimension aligns with the core Pleasantness-Comfort axis of existing models. However, its Recognisability dimension, while overlapping with the ISO's Eventfulness, distinctly prioritizes the identification of automated sound sources over general ambient richness. The partial collapse of the indoor soundscape model in this context suggests that smart home soundscape perception values informativeness and identifiability over mere sound event diversity.

The user-centered psychological perception model developed in this study can serve as a useful supplement to physically based acoustic control methods for indoor sound environments. By highlighting the perceptual roles of comfort, informativeness, and recognisability in smart home soundscapes, the study contributes to a more nuanced understanding of domestic acoustic experience in intelligent living environments. These findings provide theoretical support and design implications for the future optimisation of smart home products and domestic sound environments.

## Data Availability

The raw data supporting the conclusions of this article will be made available by the authors, without undue reservation.
