# Supplementary material for: Smart home soundscape: constructing a perceptual model with qualitative and quantitative methods
Source: Front Psychol. 2026 Jun 5;17:1803294. doi: 10.3389/fpsyg.2026.1803294 (PMC13278869; doi:10.3389/fpsyg.2026.1803294)
Supplement: Supplementary file 1 [file Data_Sheet_1.docx]

# Appendix

**Appendix A** Basic Information of Interviewees

| Demographics |  | Count | Percentage (%) |
| --- | --- | --- | --- |
| Gender | male | 10 | 35.7 |
|  | female | 18 | 64.3 |
| Education Level | High School Education | 1 | 3.6 |
|  | Associate Degree | 2 | 7.1 |
|  | Bachelor's Degree | 17 | 60.7 |
|  | Master's Degree | 6 | 21.4 |
|  | Doctoral Degree | 2 | 7.1 |
| Occupation | Student | 6 | 21.4 |
|  | Employed | 20 | 71.4 |
|  | Unemployed | 2 | 7.1 |

**Appendix B** Spectral Characteristics of the Sound Stimuli

| Category | Sound Stimuli | Spectrogram |
| --- | --- | --- |
| Indication  （50dB) | Beep sound (S1) | 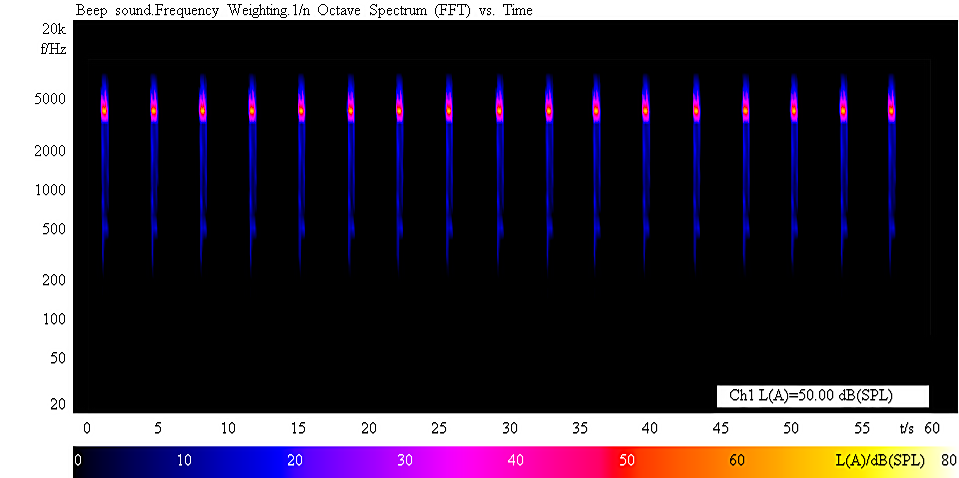 |
|  | Female voice (S2) | 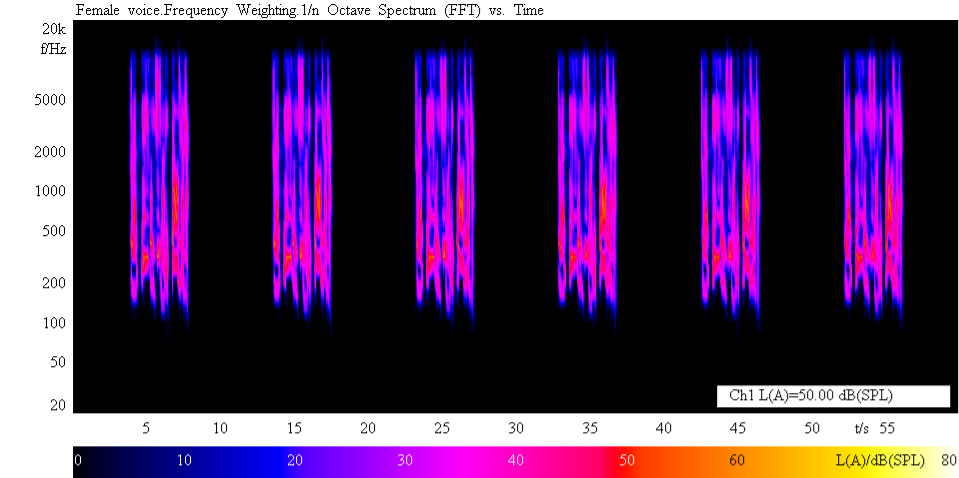 |
|  | Male voice (S3) | 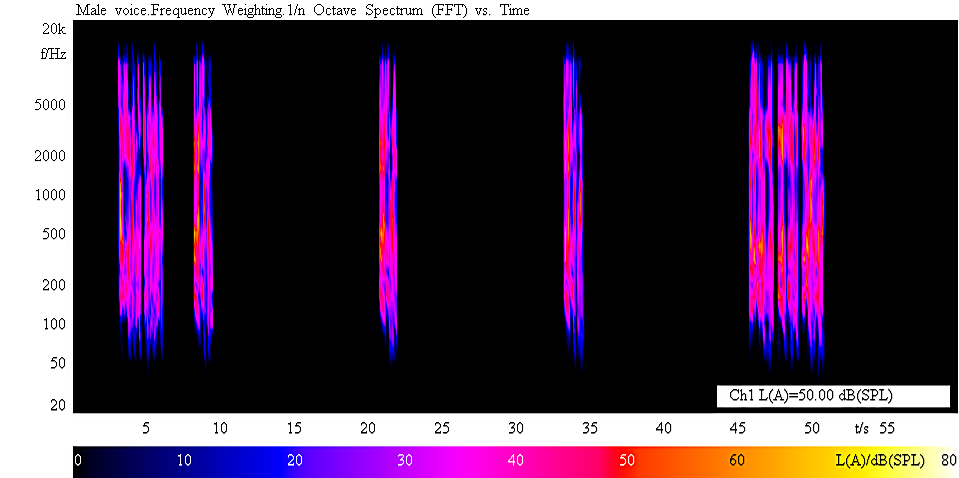 |
|  | Musical sound (S4) | 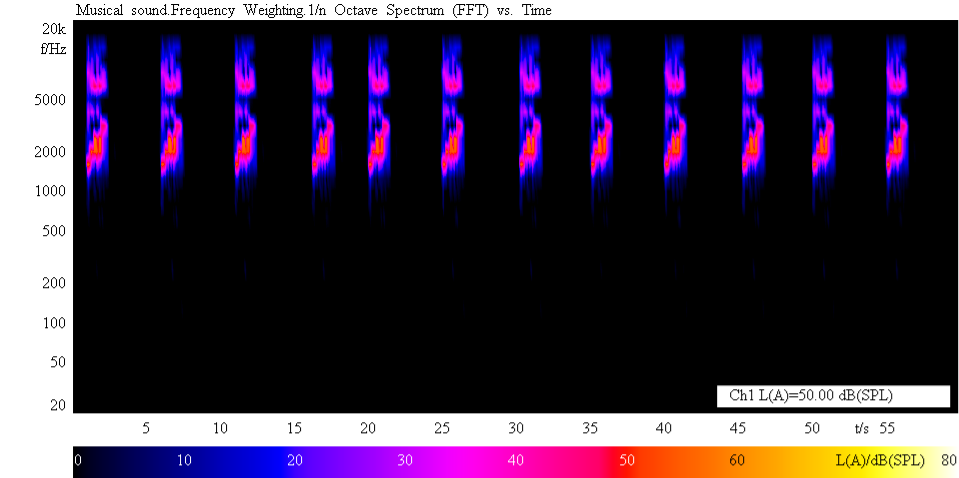 |
| Operation（40dB) | Washing machine Sound (S5) | 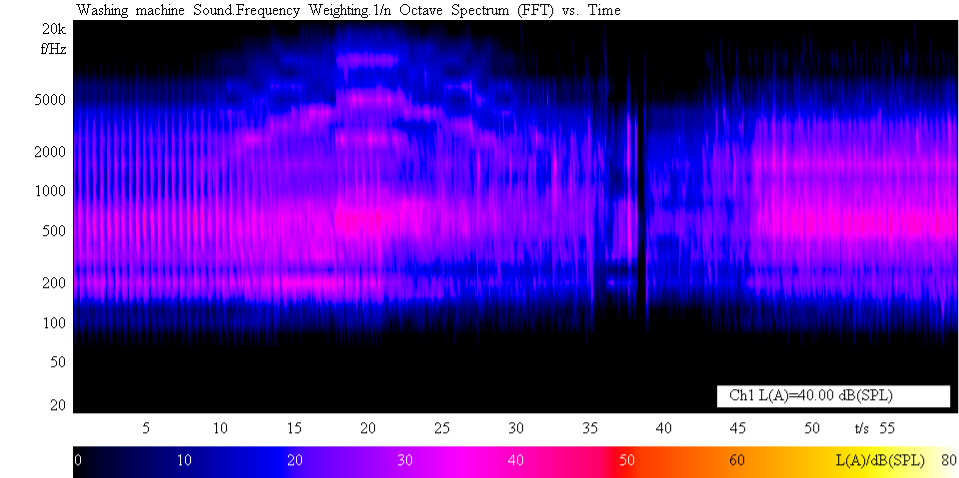 |
|  | Robotic vacuum cleaner Sound (S6) | 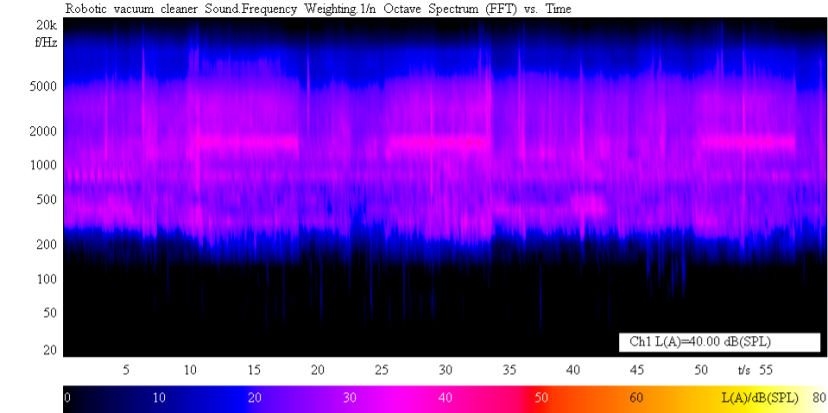 |
|  | Motorised curtain sound (S7) | 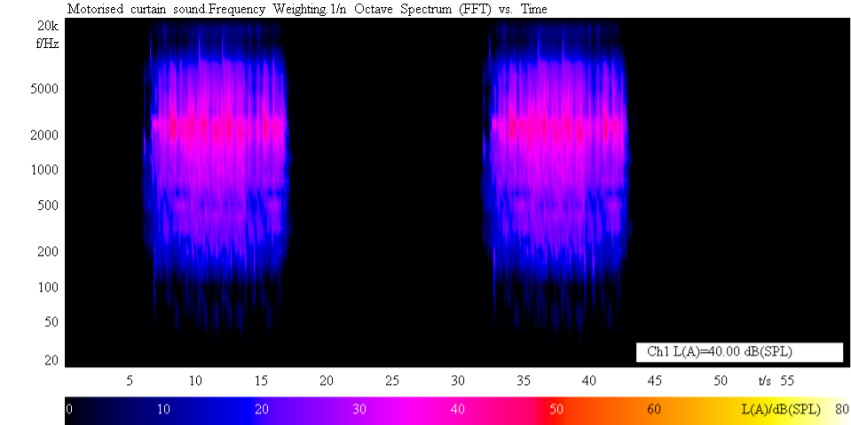 |
|  | Air conditioner sound (S8) | 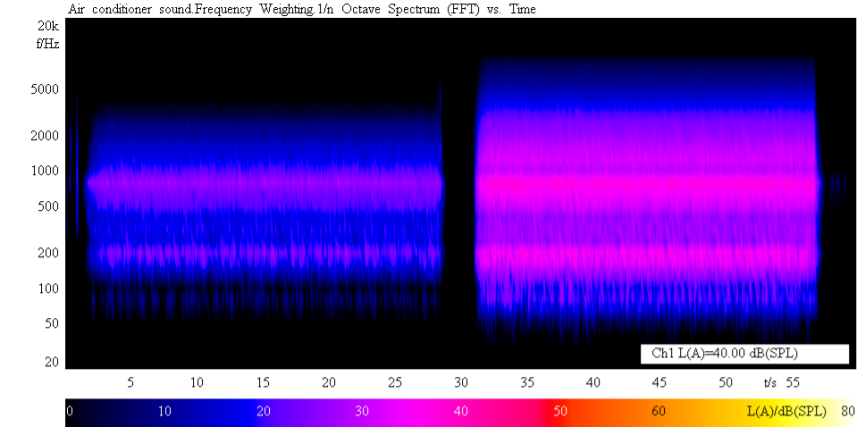 |
|  | Water purifier sound (S9) | 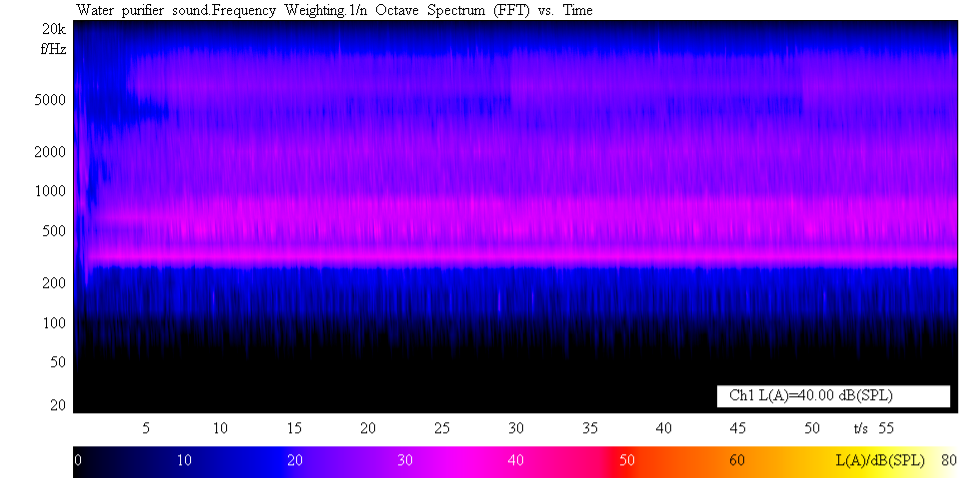 |
| Alarm（60dB) | Smoke alarm sound (S10) | 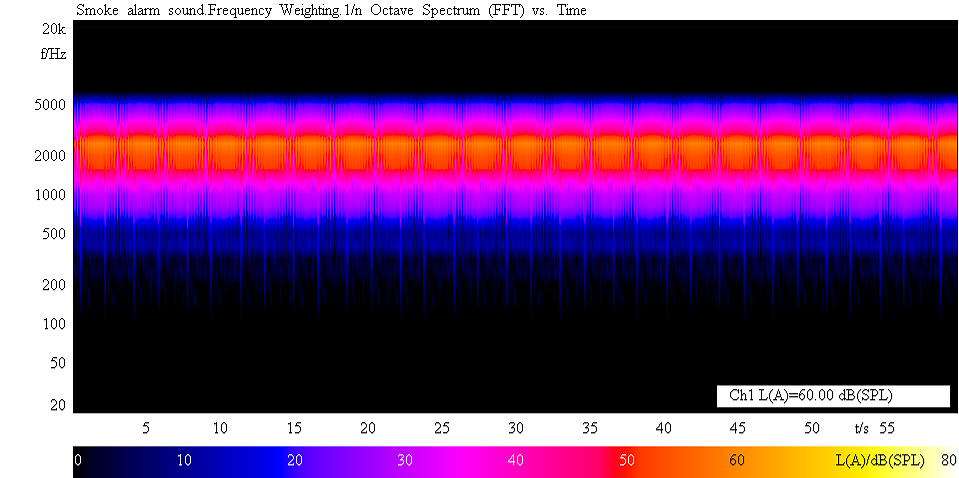 |
|  | Air purifier alarm sound (S11) | 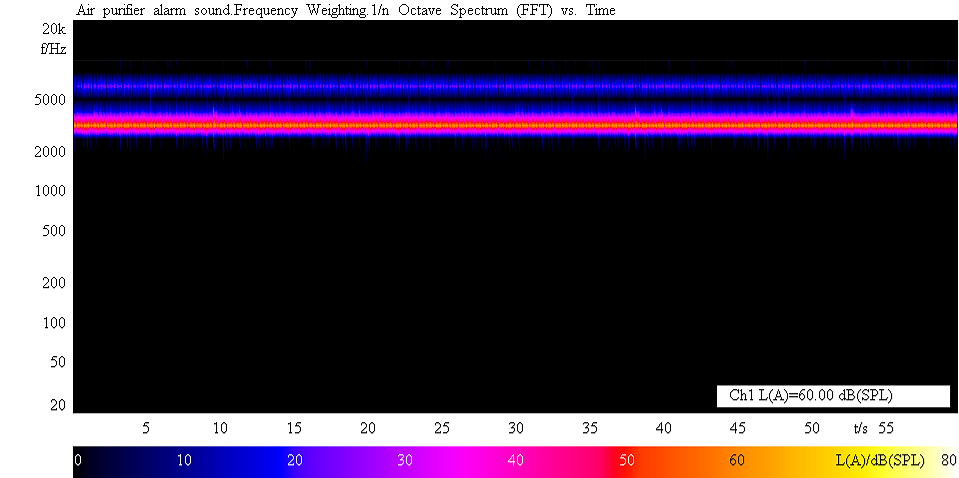 |
|  | Doorbell alarm sound (S12) | 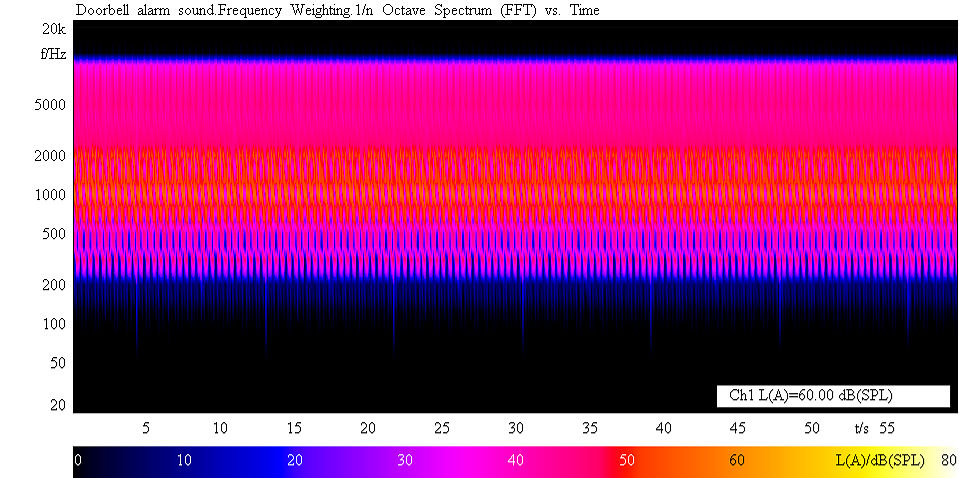 |

**Appendix C** Rotated Factor Loading Matrix for Sound Source Perception

|  | Component | | | | |
| --- | --- | --- | --- | --- | --- |
|  | 1 | 2 | 3 | 4 | 5 |
| **Rackety** | -0.855 | 0.235 | -0.153 | 0.097 | 0.157 |
| **Harsh** | -0.847 | 0.180 | -0.148 | 0.069 | -0.017 |
| **Noisy** | -0.828 | 0.231 | -0.127 | 0.139 | 0.138 |
| **Cacophonous** | -0.821 | 0.221 | -0.168 | 0.081 | 0.219 |
| **Tolerable** | 0.807 | -0.076 | 0.143 | -0.042 | -0.098 |
| **Shrill** | -0.804 | 0.101 | -0.063 | 0.049 | -0.223 |
| **Acceptable** | 0.802 | -0.079 | 0.150 | 0.014 | -0.179 |
| **Rapid** | -0.774 | 0.277 | -0.019 | -0.014 | -0.160 |
| **Soft** | 0.739 | -0.064 | 0.233 | -0.022 | 0.096 |
| **Smooth** | 0.709 | -0.178 | 0.052 | 0.047 | 0.307 |
| **Warm** | 0.679 | 0.024 | 0.336 | 0.053 | 0.004 |
| **Cold** | -0.634 | 0.103 | -0.187 | -0.050 | -0.033 |
| **Sudden** | -0.571 | 0.331 | 0.005 | -0.041 | -0.455 |
| **Predictable** | 0.544 | -0.238 | 0.048 | 0.142 | 0.431 |
| **Confusing** | -0.493 | -0.001 | 0.067 | -0.491 | 0.012 |
| **Unique** | -0.141 | 0.808 | 0.092 | -0.074 | -0.052 |
| **Distinct** | -0.216 | 0.746 | 0.114 | 0.150 | -0.001 |
| **Distinguishable** | -0.201 | 0.734 | 0.052 | 0.287 | 0.010 |
| **Meaningful** | -0.170 | 0.686 | 0.089 | 0.287 | -0.173 |
| **Informative** | -0.195 | 0.626 | 0.087 | 0.411 | -0.196 |
| **Interesting** | 0.257 | 0.099 | 0.803 | 0.012 | 0.002 |
| **Lively** | 0.205 | 0.052 | 0.793 | 0.048 | -0.087 |
| **Novel** | 0.034 | 0.218 | 0.763 | -0.053 | -0.009 |
| Euphonious | 0.473 | 0.015 | 0.682 | 0.138 | -0.055 |
| **Straightforward** | -0.016 | 0.381 | 0.048 | 0.808 | -0.022 |
| **Clear** | -0.181 | 0.357 | 0.077 | 0.776 | -0.096 |
| **Redundant** | -0.245 | -0.219 | 0.000 | -0.046 | 0.630 |
| **Deep** | 0.477 | -0.007 | -0.114 | -0.136 | 0.606 |
| **Prolonged** | -0.278 | 0.251 | -0.322 | -0.210 | 0.432 |

**Appendix D** Rotated Factor Loading Matrix for Sound Environment Perception

|  | Component | |
| --- | --- | --- |
|  | 1 | 2 |
| **Acceptable** | 0.846 | -0.086 |
| **Annoying** | -0.835 | 0.162 |
| **Oppressive** | -0.832 | 0.133 |
| **Irritating** | -0.827 | 0.123 |
| **Worried** | -0.826 | 0.17 |
| **Tolerable** | 0.822 | -0.095 |
| **Comfortable** | 0.82 | -0.181 |
| **Belonging** | 0.8 | -0.169 |
| **Startling** | -0.797 | 0.176 |
| **Customary** | 0.794 | -0.183 |
| **Companionable** | 0.778 | -0.202 |
| **Pleasant** | 0.778 | -0.081 |
| **Safe** | 0.775 | -0.137 |
| **Homely** | 0.756 | -0.124 |
| **Confusing** | -0.71 | 0.051 |
| **Chaotic** | -0.705 | -0.145 |
| **Orderly** | 0.585 | 0.182 |
| **Separate** | -0.111 | 0.839 |
| **Indistinguishable** | 0.005 | -0.819 |
| **Recognisable** | -0.017 | 0.806 |
| **Harmonised** | 0.32 | -0.587 |
